# Exploring C-To-G Base Editing in Rice, Tomato, and Poplar

**DOI:** 10.3389/fgeed.2021.756766

**Published:** 2021-09-15

**Authors:** Simon Sretenovic, Shishi Liu, Gen Li, Yanhao Cheng, Tingting Fan, Yang Xu, Jianping Zhou, Xuelian Zheng, Gary Coleman, Yong Zhang, Yiping Qi

**Affiliations:** ^1^ Department of Plant Science and Landscape Architecture, University of Maryland, College Park, MD, United States; ^2^ Department of Biotechnology, School of Life Science and Technology, University of Electronic Science and Technology of China, Center for Informational Biology, Chengdu, China; ^3^ Institute for Bioscience and Biotechnology Research, University of Maryland, Rockville, MD, United States

**Keywords:** C-to-G base editors, PAM-less, SPRY, rice, tomato, poplar

## Abstract

As a precise genome editing technology, base editing is broadly used in both basic and applied plant research. Cytosine base editors (CBEs) and adenine base editors (ABEs) represent the two commonly used base editor types that mediate C-to-T and A-to-G base transition changes at the target sites, respectively. To date, no transversion base editors have been described in plants. Here, we assessed three C-to-G base editors (CGBEs) for targeting sequences with SpCas9’s canonical NGG protospacer adjacent motifs (PAMs) as well as three PAM-less SpRY-based CGBEs for targeting sequences with relaxed PAM requirements. The analyses in rice and tomato protoplasts showed that these CGBEs could make C-to-G conversions at the target sites, and they preferentially edited the C6 position in the 20-nucleotide target sequence. C-to-T edits, insertions and deletions (indels) were major byproducts induced by these CGBEs in the protoplast systems. Further assessment of these CGBEs in stably transformed rice and poplar plants revealed the preference for editing of non-GC sites, and C-to-T edits are major byproducts. Successful C-to-G editing in stably transgenic rice plants was achieved by rXRCC1-based CGBEs with monoallelic editing efficiencies up to 38% in T0 lines. The UNG-rAPOBEC1 (R33A)-based CGBE resulted in successful C-to-G editing in polar, with monoallelic editing efficiencies up to 6.25% in T0 lines. Overall, this study revealed that different CGBEs have different preference on preferred editing sequence context, which could be influenced by cell cycles, DNA repair pathways, and plant species.

## Introduction

Since 2016, numerous CRISPR-Cas9-derived base editors have been reported and were first used to edit mammalian genomes, and more recently for editing plant genomes (Molla and Yang, 2019; Zhang et al., 2019; Gurel et al., 2020). Currently, there are two major types of base editors used to edit plant genomes. The first type is cytosine base editors (CBEs) which direct C-to-T transition base changes (Komor et al., 2016; Nishida et al., 2016). Many CBEs based on different cytidine deaminases were reported for use in plants including rABOBEC1 (Li et al., 2017; Lu and Zhu, 2017; Zong et al., 2017), PmCDA1 (Shimatani et al., 2017; Tang et al., 2019; Zhong et al., 2019), hAID (Ren et al., 2018), human APOBEC3A (A3A) (Zong et al., 2018; Cheng et al., 2021), APOBEC3B (A3B) (Jin et al., 2020), and A3A/Y130F (Li et al., 2021a; Ren et al., 2021a; Randall et al., 2021). The second type is adenine base editors (ABEs) which confer A-to-G transition base changes (Gaudelli et al., 2017). Unlike CBEs, ABEs utilize artificially evolved adenosine deaminases which showed high-efficiency and high-purity A-to-G base conversions in human cells (Gaudelli et al., 2017; Richter et al., 2020) and plants at both canonical NGG PAM sites and relaxed PAM sites (Hua et al., 2018; Li et al., 2018; Yan et al., 2018; Li et al., 2021b; Ren et al., 2021b; Xu et al., 2021; Yan et al., 2021). The development of plant CBEs and ABEs, while largely based on reagents first developed in human cells, has generated relatively high editing efficiency in many plant species and greatly boosted genome editing applications in agriculture (Molla and Yang, 2019; Zhang et al., 2019; Gao, 2021).

CBEs and ABEs can make C-to-T (G-to-A in the reverse complementary strand) and A-to-G (T-to-C in the reverse complementary strand) edits, respectively. They only induce base transition changes and collectively render 4 out of 12 possible base substitutions. It would be highly desirable to develop base editors that can perform transversion base changes (pyrimidine to purine or purine to pyrimidine). Although it is not uncommon to observe C-to-G editing events with CBEs, achieving C-to-G editing at higher efficiency requires dedicated C-to-G base editors. Excitingly, several C-to-G base editors were reported in human cells recently (Chen et al., 2021; Kurt et al., 2021; Zhao et al., 2021). These C-to-G base editors (CGBEs) are composed of a nCas9 nickase, a cytidine deaminase rAPOBEC1 (Chen et al., 2021; Zhao et al., 2021) or its engineered form rAPOBEC1 (R33A) (Kurt et al., 2021) that showed reduced off-target effects at the genome and transcriptome levels in human cells (Grunewald et al., 2019; Doman et al., 2020), and a base excision repair (BER) protein such as a uracil DNA glycosylase sourced from *E. coli* (UNG) (Kurt et al., 2021; Zhao et al., 2021) or rXRCC1 sourced from rat (Chen et al., 2021). The editing efficiency of these CGBEs is highly target-dependent and they all prefer a narrow editing window centered on the cytosine at the sixth position (C6) of the target sequences (Chen et al., 2021; Kurt et al., 2021; Zhao et al., 2021).

Such CGBEs hold great promise for C-to-G base editing in plants, further expanding the genome engineering revolution in agriculture (Molla et al., 2020). Since many of the CBEs and ABEs that showed promising editing performance in human cells were later found to be also highly efficient base editors in plants, we reasoned that development of plant CGBEs based on the human cell-tested or proven CGBEs would represent a straightforward approach to establish a first-generation plant C-to-G base editing tools. Therefore, in this study we set out to closely compare the three top CGBE platforms (Chen et al., 2021; Kurt et al., 2021; Zhao et al., 2021) with optimization for plant delivery and expression. To have a broad implication in tool development, we assessed the CGBEs in three distinct plant species, including rice (an annual monocot), tomato (an annual dicot), and poplar (a perennial dicot tree). By doing so, we hope to gain a better understanding of possible editing outcomes for these CGBEs among different plant species and cell types. As a result, the knowledge gained through this study could further guide future optimization toward achieving highly efficient C-to-G base editing in plants.

## Materials and Methods

### Vector Construction

All the primers used in this study are listed in Supplementary Table S1. The pYPQ265 vector (Addgene # 164712) was reported in our recent publication (Ren et al., 2021a). To prepare Gateway compatible attL1-attR5 entry clone pYPQ265K (Addgene #173997), the backbone obtained from pYPQ166-D10A plasmid after restriction digestion with BsrGI-HF (NEB, catalog # R3575*) and NcoI-HF (NEB, catalog # R3193*) and CGBE1-gBk synthetic DNA (IDT gBlock) digested with BsrGI-HF and NcoI-HF were ligated together. Gateway compatible attL1-attR5 entry clone pYPQ265L2 (Addgene #174000) was prepared using NEBuilder® HiFi DNA Assembly kit (NEB, catalog # E5520) with primers 266E-INS_fwd and 266E-INS_rev to amplify zCas9-SpRY from pYPQ166-SpRY (Addgene # 161,520) and primers 266E-BB_fwd and 266E-BB_rev to amplify backbone from pYPQ265K. Gateway compatible attL1-attR5 entry clone pYPQ265N1 (Addgene #173998) was also prepared using NEBuilder® HiFi DNA Assembly kit with primers 265N1-BB_fwd and 265N1-BB_rev to amplify backbone from pYPQ265 and UNG-gBk synthetic DNA (IDT gBlock). Gateway compatible attL1-attR5 entry clone pYPQ265N2 (Addgene #174001) was prepared using NEBuilder® HiFi DNA Assembly kit with primers 266E-INS_fwd and 266E-INS_rev to amplify zCas9-SpRY from pYPQ166-SpRY and primers 266E-BB_fwd and 266E-BB_rev to amplify backbone from pYPQ265N1. Gateway compatible attL1-attR5 entry clone pYPQ265O1 (Addgene #173999) was prepared using NEBuilder® HiFi DNA Assembly kit with primers 265O1-BB_fwd and 265O1-BB_rev to amplify backbone from pYPQ265 and rXRCC1-gBk synthetic DNA (IDT gBlock). Gateway compatible attL1-attR5 entry clone pYPQ265O2 (Addgene #174002) was prepared using NEBuilder® HiFi DNA Assembly kit with primers 266E-INS_fwd and 266E-INS_rev to amplify zCas9-SpRY from pYPQ166-SpRY and primers 266E-BB_fwd and 266E-BB_rev to amplify backbone from pYPQ265O1.

All the T-DNA vectors used in this study are listed in Supplementary Table S2 and were constructed using Gateway LR assembly reactions based on the protocols described previously (Lowder et al., 2015). To prepare sgRNA entry clones, forward and reverse primers (Supplementary Table S1) were phosphorylated with T4 polynucleotide kinase (NEB, catalogue #M0201*), annealed, and ligated with T4 DNA ligase (NEB, catalogue #M0202*) into pYPQ141C (Addgene # 69292) or pYPQ141D (Addgene # 69293) for rice base editing, and into pYPQ141B (Addgene #69291) for poplar and tomato base editing. Individual Gateway LR reactions consisted of an attL5-attL2 sgRNA entry clone, an attL1-attR5 base editor entry clone, and an attR1-attR2 destination vector. For rice base editing, the destination vector was pYPQ203 (Addgene # 86207) containing ZmUBI promotor for base editor expression. For tomato base editing, the destination vector was pCGS710 containing 2x35S promoter. For poplar base editing, the destination vector was pYPQ202 (Addgene # 86198) containing AtUBQ10 promoter. The names of T-DNA vectors resulted from this LR Gateway assembly start with “pLR” (Supplementary Table S2). Both sgRNA and base editor entry clone recombination regions were confirmed by Sanger sequencing. Final T-DNA vectors were confirmed by restriction digestion with EcoRV-HF (NEB, catalog # R3195*) for T-DNAs used in tomato and with EcoR1-HF (NEB, catalog # R3101*) for T-DNAs used in rice and poplar.

### Rice Protoplast Transformation and Stable Transformation

The Japonica cultivar Kitaake rice were used. The rice protoplast transformation was done by following our previously published protocols (Tang et al., 2017; Ren et al., 2019; Zhong et al., 2020). The rice stable transformation based on Agrobacterium was done by following a previously published protocol (Zhou et al., 2017; Zhou et al., 2019). Genomic DNA from protoplasts and transgenic seedlings were extracted using the CTAB method (Stewart and Via, 1993).

### Tomato Protoplast Transformation

The Micro Tom Tomato cultivar was used. The tomato protoplast transformation was performed according to a recent publication (Randall et al., 2021). Transformed tomato protoplasts were directly mixed with Phire Plant Direct PCR Master Mix (ThermoFisher) for the downstream PCR based analysis. PCR products were pooled together for next-generation sequencing (Genewiz, United States).

### Poplar Stable Transformation

Populus alba x tremula clone 717-1B4 was used for stable transformation as described (Leple et al., 1992). Transformed shoots were selected by regenerating on media containing hygromycin. The rooted plants were propagated and used for further genotyping. Two rounds Hi-Tom PCR were preceded to obtain amplicons using Phire Plant Direct PCR Master Mix (ThermoFisher).

### Mutagenesis Analysis

For analysis of genome editing in rice and tomato protoplasts, barcoded PCR amplicons were subjected to NGS using an Illumina HiSeqX platform. The resulting data were analyzed by CRISPRMatch (You et al., 2018). For analysis of genome editing in stably transformed T0 lines in rice, PCR amplicons covering each target site were used for Sanger Sequencing followed by decoding. For analysis of genome editing in stably transformed T0 lines in poplar, barcoded PCR amplicons were sequenced by an Illumina HiSeqX platform (Genewiz, United States), followed by analysis using the HiTom tool (Liu et al., 2019) and CRISPRMatch (You et al., 2018).

## Results

### Development and Comparison of Three CGBEs in Rice Protoplasts

**FIGURE 1:**
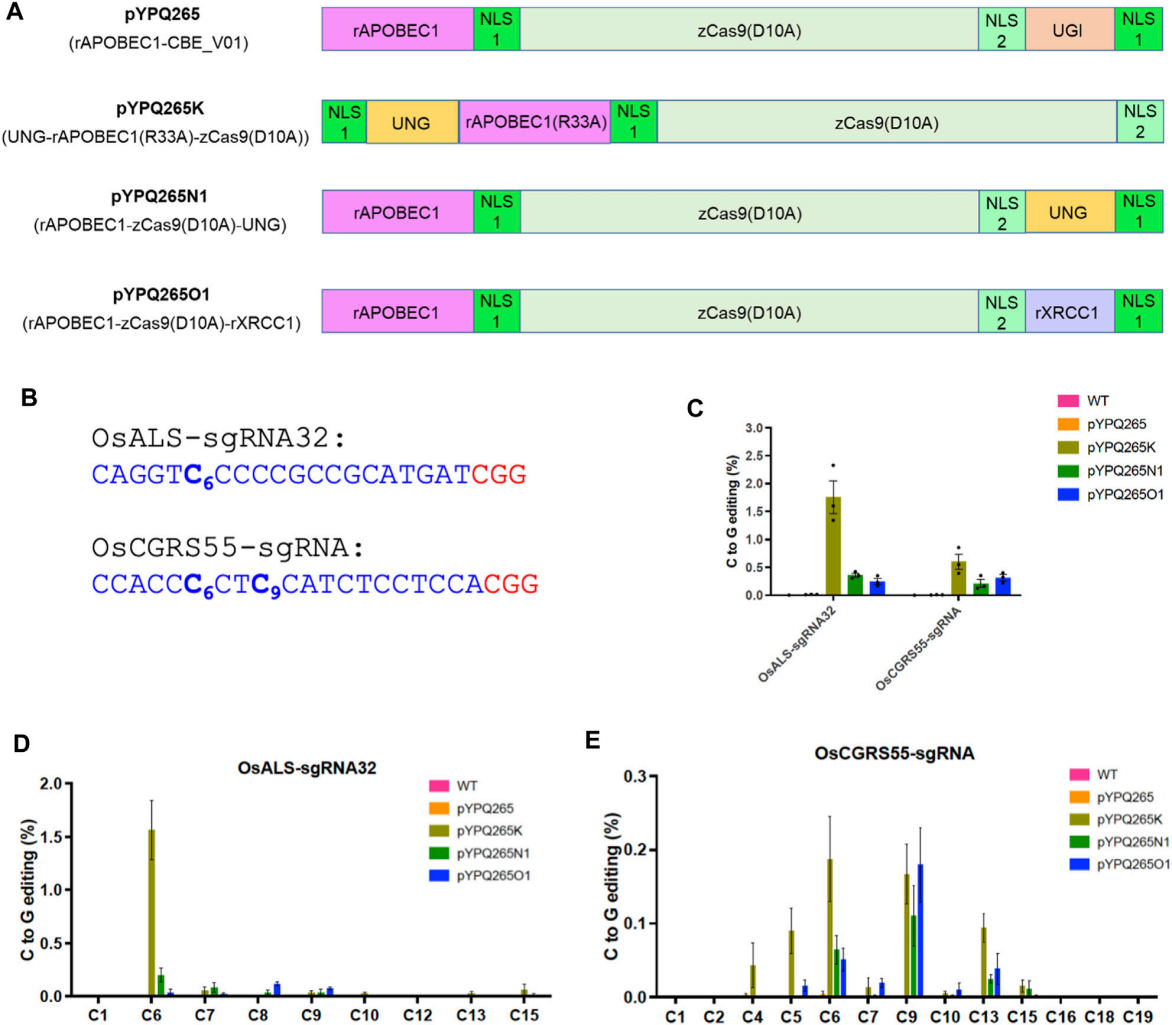
Assessment of BE3 and three CGBEs in rice protoplasts. **(A)** Diagram of BE3 and three CGBEs. Note each nuclear localization single (NLS) is indicated by a green box. NLS 1 is a monopartite SV40 nuclear localization signal and NLS 2 is a bipartite nuclear localization signal of nucleoplasmin. Both NLS1 and NLS2 are recognized by importin α. **(B)** The target sites in the rice genome. The protospacer sequence is highlighted in blue and the PAM is highlighted in red. **(C)** NGS quantification of C-to-G editing by four base editors in rice protoplasts. For the wild type (WT) samples, sterile deionized water was used in protoplast transformation. **(D)** NGS analysis of C-to-G editing windows by different base editors at the OsALS-sgRNA32 site. **(E)** NGS analysis of the C-to-G editing windows by different base editors at the OsCGRS55-sgRNA site. The error bars represent standard errors of three biological replicates.

To develop plant CGBEs, we decided to compare the best performing CGBEs from the three recent studies used to edit in human cells (Chen et al., 2021; Kurt et al., 2021; Zhao et al., 2021). Since these CGBEs were all based on rAPOBEC1, the rAPOBEC1-based CBE-BE3 (pYPQ265, BE3) (Ren et al., 2021a) was included as a control (Figure 1A). We used a maize codon optimized Cas9 (zCas9) which was previously shown to be very efficient for genome editing in Arabidopsis (Wang et al., 2015), maize (Lee et al., 2019), and wheat (Li et al., 2021c), and recently used for efficient base editing in rice (Ren et al., 2021a; Ren et al., 2021b), tomato (Randall et al., 2021), and poplar (Li et al., 2021a). We applied rice codon optimization for the other components of these CGBEs and generated three Gateway entry clones for them, which are pYPQ265K with UNG-rAPOBEC1 (R33A) fusion to the N-terminus of nCas9, pYPQ265N1 with rAPOBEC1 and UNG fusion to both ends of nCas9, and pYPQ265O1 with rAPOBEC1 and xRCC1 fusion to both ends of nCas9 (Figure 1A). These vectors are compatible with our multiplexed CRISPR-Cas9 toolbox which can generate T-DNA expression vectors in a single step three-way Gateway LR reaction (Lowder et al., 2015).

We first assessed these CGBEs in rice. Two target sites (OsALS-sgRNA32 and OsCGRS55-sgRNA) were chosen, with both containing multiple cytosines in the target sequences, allowing for assessment of editing efficiency at individual cytosines (Figure 1B). The single guide RNAs (sgRNAs) were expressed under an OsU3 or OsU6 promoter, while the CGBE protein fusions were expressed under a maize ubiquitin promoter (ZmUbi). We compared the three CGBEs with BE3 in rice protoplasts. The editing outcomes were analyzed by next-generation sequencing (NGS) of polymerase chain reaction (PCR) amplicons. The data showed no detectable C-to-G base editing by the canonical BE3 (pYPQ265) (Figure 1C), which rather generated high levels of C-to-T base editing at both sites, ∼7% at the OsALS-sgRNA32 site and ∼13% at the OsCGRS55-sgRNA site (Supplementary Figure S1A). The data indicate that BE3 only generates C-to-T base editing, not C-to-G base editing. By contrast, all three CGBEs showed detectable C-to-G base editing, with pYPQ265K outperforming pYPQ265N1 and pYPQ265O1 (Figure 1C). pYPQ265K generated ∼1.75% C-to-G editing frequency at the OsALS-sgRNA32 site and ∼0.70% editing frequency at the OsCGRS55-sgRNA site, while pYPQ265N1 and pYPQ265O1 generated 0.25–0.40% C-to-G editing frequencies (Figure 1C). All three CGBEs could edit multiple cytosines in the target sequences, with high C-to-G conversion activity for C6 in the target sequences (Figures 1D,E). Interestingly, while pYPQ265K showed relatively high C-to-G editing at both C6 and C9 positions at the OsCGRS55-sgRNA site, pYPQ265N1 and pYPQ265O1 showed a preference for editing the C9 position at this target site (Figure 1E).

We also examined other editing outcomes by the three CGBEs at the two target sites. Relatively high levels of C-to-T base editing were observed for pYPQ265O1, ∼4% at the OsALS-sgRNA32 site and ∼3% at the OsCGRS55-sgRNA site, while pYPQ265N1 showed minimal C-to-T editing at these sites (Supplementary Figure S1A). The C-to-T editing window for BE3 is C4-C10 (Supplementary Figures S1B,C), consistent with previous reports (Komor et al., 2016). By contrast, rather low C-to-A editing frequencies were detected for all base editors (Supplementary Figure S2). Interestingly, high levels of insertions and deletions (indels) were generated by all three CGBEs, but not by BE3, with pYPQ265K showing the highest (∼12% at both target sites) (Supplementary Figure S3), which could be attributed to the removal of UGI in these editors. Together, these data suggest C-to-T edits and indels are major byproducts of these CGBEs in rice protoplasts.

### Comparison of Three CGBEs in Tomato Protoplasts

**FIGURE 2:**
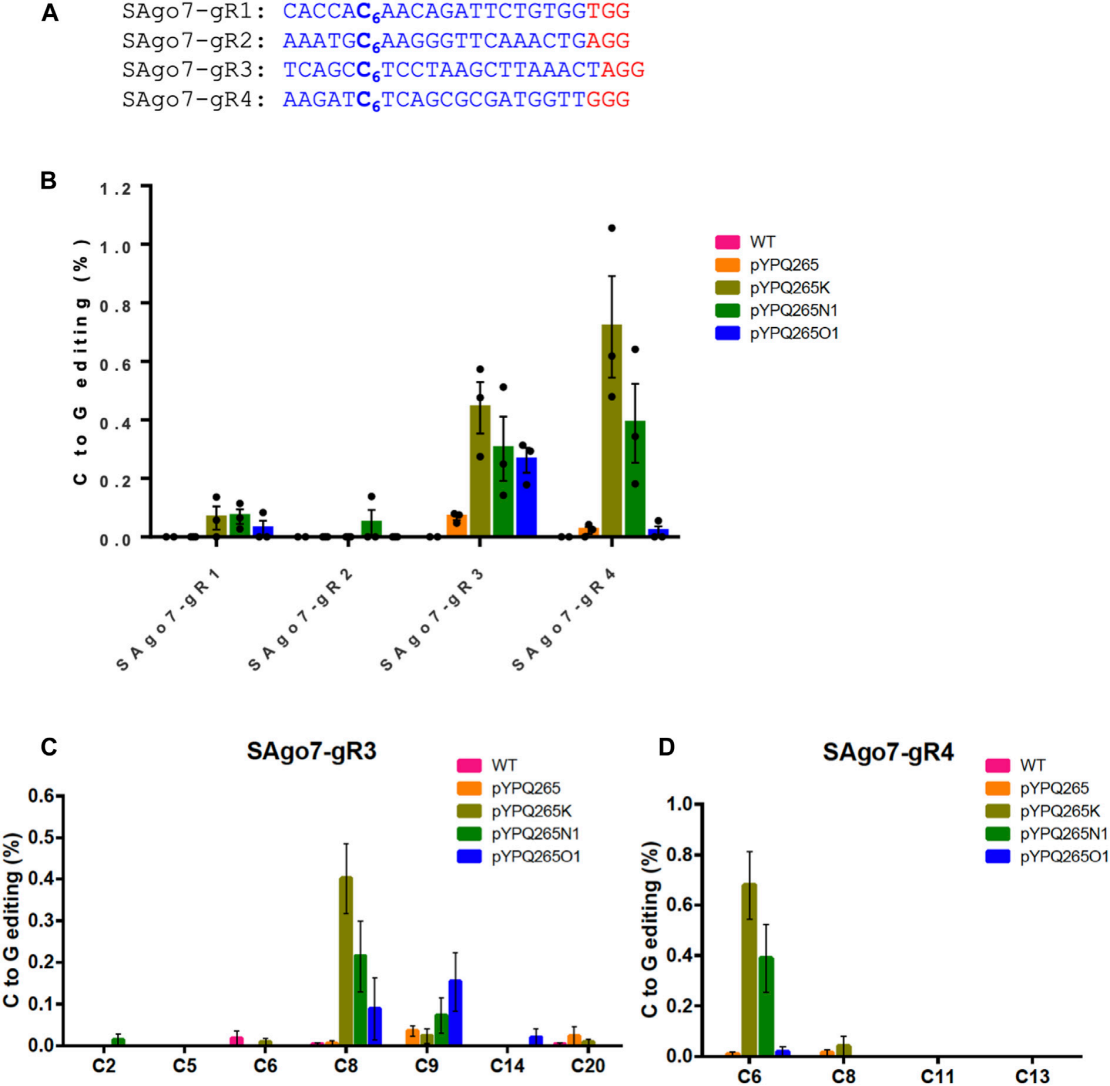
Assessment of BE3 and three CGBEs in tomato protoplasts **(A)** Four target sites in the tomato genome. The PAM sequences are underlined and highlighted in red. **(B)** NGS quantification of C-to-G editing by four base editors in tomato protoplasts. For the WT samples, water was used in protoplast transformation. **(C, D)** NGS analysis of editing windows by different base editors at SAgo7-gR3 and SAgo7-gR4 target sites. The error bars represent standard errors of three biological replicates.

We next assessed these CGBEs in tomato protoplasts. Four target sites were chosen in the Solanum lycopersicum AGO7 (SAG O 7) gene (Husbands et al., 2009) (Figure 2A). We expressed the sgRNAs under the AtU3 promoter and CGBE protein fusions under the 2 × 35S promoter. These three CGBEs were also compared with BE3 in the tomato protoplasts. The editing outcomes were analyzed by NGS of PCR amplicons. While the CGBEs mostly failed at editing the SAgo7-gR1 and SAgo7-gR2 sites, they showed 0.3–0.7% C-to-G base editing frequencies at the SAgo7-gR3 and SAgo7-gR4 sites with pYPQ265K showing the overall higher C-to-G editing frequencies (Figure 2B). As expected, the BE3 pYPQ265 failed to covert C-to-G changes at all four target sites (Figure 2B). Analysis of editing windows showed different editing preference at the various sites. C8 was preferred by pYPQ265K and pYPQ265N1, and C9 was preferred by pYPQ265O1 at the SAgo7-gR3 site (Figure 2C), while C6 was preferred by pYPQ265K and pYPQ265N1 at the SAgo7-gR4 site (Figure 2D). These data suggest sequence context-dependent C-to-G editing by these CGBEs in tomato. Analysis of other editing outcomes showed that C-to-T editing and indels are major byproducts, ranging from ∼2 to ∼8% (Supplementary Figures S4, S5), while C-to-A editing was no more than 0.3% at all target sites (Supplementary Figure S6). These tomato protoplast data were generally consistent with the rice protoplast data.

### Development and Assessment of Three SpRY-Based CGBEs in Rice Protoplasts

**FIGURE 3:**
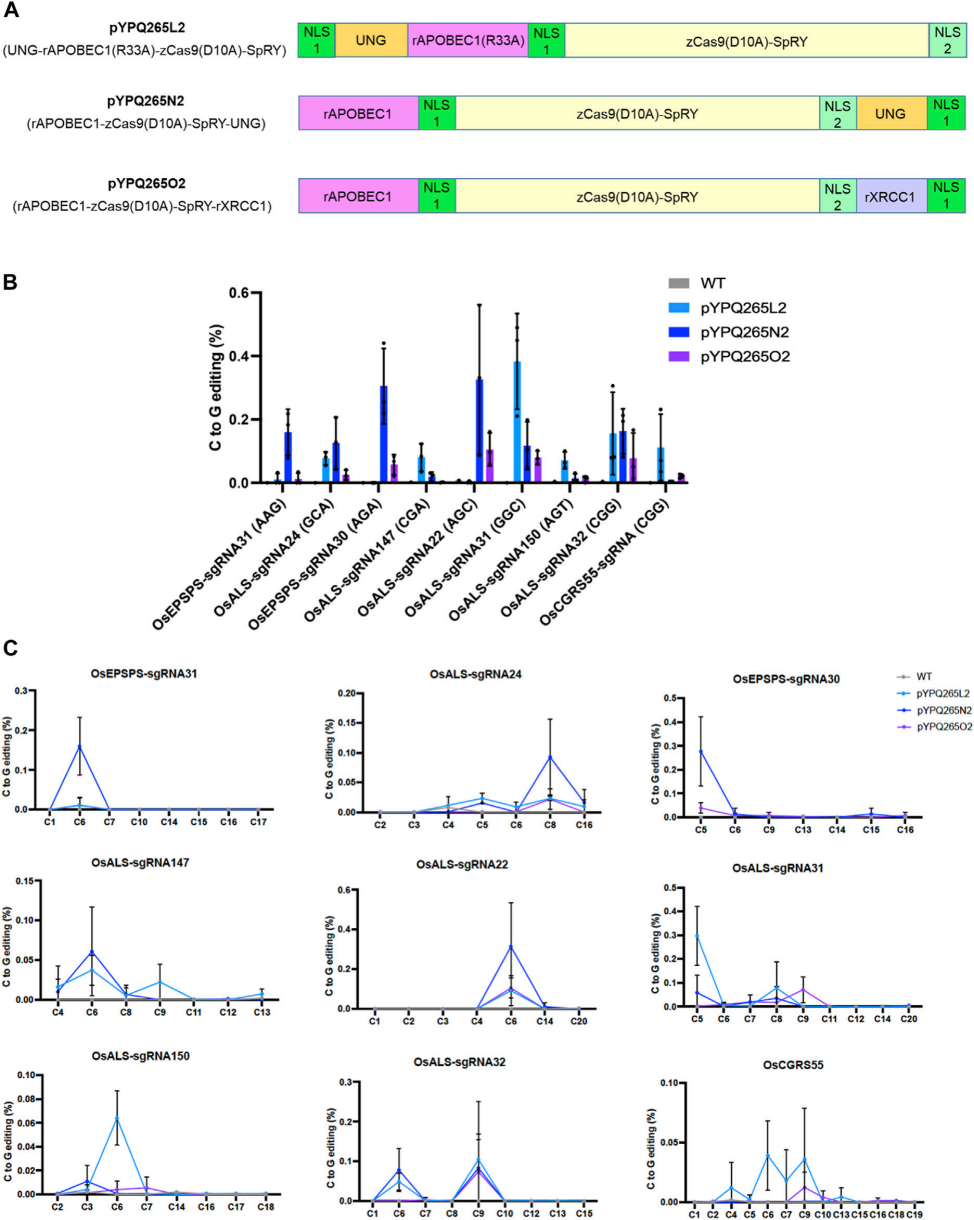
Assessment of three SpRY-based CGBEs in rice protoplasts. **(A)** Diagram of three SpRY-based CGBEs. Note each NLS is indicated by a green box. NLS 1 is a monopartite SV40 nuclear localization signal and NLS 2 is a bipartite nuclear localization signal of nucleoplasmin. Both NLS1 and NLS2 are recognized by importin α. **(B)** NGS quantification of C-to-G editing at nine target sites in the rice genomes. **(C)** NGS analysis of editing windows by different SpRY-based CGBEs across different target sites. The error bars represent standard errors of three biological replicates.

The C-to-G base editing data from rice and tomato protoplasts suggest that different CGBEs favor different cytosine positions in the target sites. To accommodate flexible editing at the possible favorable cytosines in the target sequences, we generated three corresponding CGBEs based on PAM-less SpRY (Walton et al., 2020; Li et al., 2021b; Ren et al., 2021b; Xu et al., 2021), namely pYPQ265L2, pYPQ265N2, and pYPQ265O2 (Figure 3A). We targeted seven relaxed NNN PAM sites as well as two NGG PAM sites that we targeted earlier with the wild type (WT) nCas9. Since these CGBEs prefer C6 in the 20-nucleotide targets in human cells (Chen et al., 2021; Kurt et al., 2021; Zhao et al., 2021), we made sure all these nine target sites contained a cytosine at the sixth position. Among the nine target sites, C-to-G editing was detectable (at ∼0.1% or higher) at six sites (OsALS-sgRNA24, OsALS-sgRNA147, OsALS-sgRNA22, OsALS-sgRNA31, OsALS-sgRNA32, and OsCGRS55-sgRNA) by pYPQ265L2, at six sites (OsEPSPS-sgRNA31, OsALS-sgRNA24, OsEPSPS-sgRNA30, OsALS-sgRNA22, OsALS-sgRNA31, and OsALS-sgRNA32) by pYPQ265N2, and at three sites (OsALS-sgRNA22, OsALS-sgRNA31, and OsALS-sgRNA32) by pYPQ265O2 (Figure 3B). Analysis of editing windows regardless of the editor showed that the highest editing was observed at C6 at six target sites (OsEPSPS-sgRNA31, OsALS-sgRNA147, OsALS-sgRNA22, OsALS-sgRNA150, OsALS-sgRNA32, and OsCGRS55-sgRNA). Occasionally, C8 (e.g., at the OsALS-sgRNA24 site) was favored or C9 (e.g., at the OsALS-sgRNA32 and OsCGRS55-sgRNA) was co-favored with C6 for C-to-G editing (Figure 3C). The three SpRY-based CGBEs showed variable editing frequencies at these preferred editing positions, suggesting their different sequence preference for C-to-G editing.

We also assessed the byproduct editing outcomes by these SpRY-based CGBEs. Interestingly, pYPQ265O2 showed relatively higher levels of C-to-T editing (>1%) at three target sites (OsEPSPS-sgRNA30, OsALS-sgRNA22, and OsALS-sgRNA31), while pYPQ265L2 and pYPQ265N2 displayed low C-to-T editing frequencies (Supplementary Figure S7A). These C-to-T editing events appeared to have a larger editing window (C4-C8), even though peak editing frequencies were also often found to be centered around C6 (Supplementary Figure S7B). Indel frequencies with ∼1–4% were generated by pYPQ265L2 and pYPQ265N2 at three target sites (OsALS-sgRNA22, OsALS-sgRNA31, and OsCGRS55-sgRNA) (Supplementary Figure S8). C-to-A base editing frequencies by these SpRY-based CGBEs were very low at all target sites, which were close to the background level of the negative controls (Supplementary Figure S9). These data showed that C-to-T editing and indels are also common byproducts of the three SpRY-based CGBEs in rice protoplasts.

### Assessments of CGBEs in Stable Rice Lines

**FIGURE 4:**
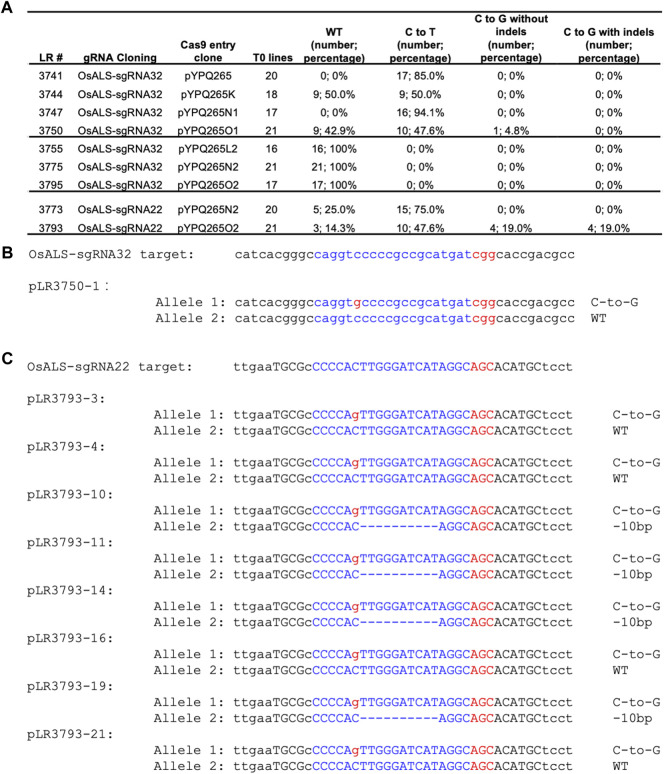
C-to-G base editing in stable rice lines **(A)** Summary of editing outcomes in transgenic T0 lines by different base editors. In brackets, number corresponds to the number of T0 lines having defined editing outcomes. **(B)** An example T0 line with a pure (i.e., monoallelic and non-chimeric). C-to-G editing allele **(C)** Example T0 lines with pure (i.e., monoallelic and non-chimeric). C-to-G editing alleles. The target sequences are highlighted in blue. The PAM sequences and the C-to-G changes are highlighted in red.

After development and assessment of these CGBEs in protoplasts, we sought to test them in stably transformed rice plants. We chose the OsALS-sgRNA32 site because it was targeted by all six CGBEs and the control BE3 in rice protoplasts. The seven T-DNA constructs corresponding to these seven base editors were used for Agrobacterium-mediated transformation of rice. We genotyped 16 to 21 individual T0 lines to reveal editing outcome at this target site for these constructs. High C-to-T base editing (47.6–94.1%) was observed for canonical BE3 (pYPQ265) and three CGBEs recognizing the canonical NGG PAMs (pYPQ265K, pYPQ265N1, and pYPQ265O1) (Figure 4A). Only pYPQ265O1 generated one monoallelic C-to-G editing at the C6 position (Figures 4A,B). The SpRY-based CGBEs failed to generate any editing events at the OsALS-sgRNA32 site among the 16–21 T0 transgenic lines examined (Figure 4A). We decided to test PAM-less C-to-G editing at the OsALS-sgRNA22 site with a relaxed AGC PAM. Our earlier rice protoplast data showed that C-to-G editing was observed for pYPQ265N2 and pYPQ265O2 (Figure 3B). Analysis of transformed rice lines showed quite high frequency C-to-T editing, 75.0% for pYPQ265N2 and 47.6% for pYPQ265O2 (Figure 4A). Importantly, four T0 lines (pLR3793-3, 4, 16, and 21) carried monoallelic C-to-G editing at the C6 position and four additional T0 lines (pLR3793-10, 11, 14, and 19) carried biallelic editing events each containing one C-to-G editing allele at the C6 position with the other allele being 10bp deletion (Figure 4C). Altogether, these data suggest that the rXRCC1-based CGBEs (pYPQ265O1 and pYPQ265O2) could generate pure C-to-G editing at the C6 position of the target sequences in rice stable lines. Since OsALS encodes an essential enzyme, complete knockout of OsALS would be lethal. Hence, it is likely the editing frequencies that we observed at OsALS were underestimated.

### Assessment of CGBEs in Stable Poplar Lines

**FIGURE 5:**
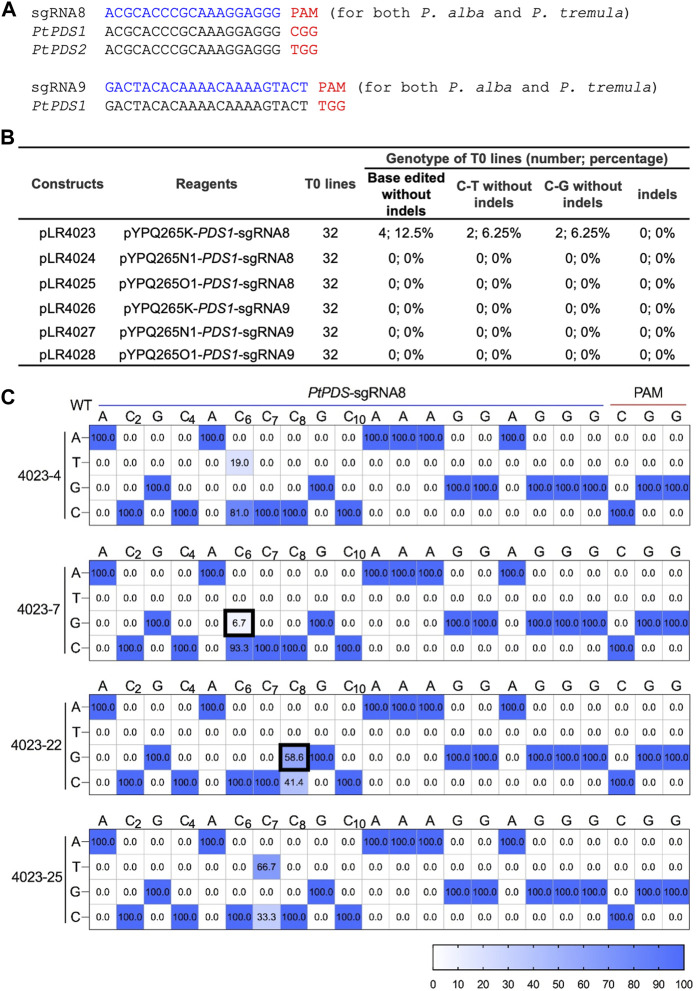
C-to-G base editing in stable poplar lines **(A)** Target sequences in the poplar hybrid. Note both *P. alba* and *P. tremula* genomes are targetable by the sgRNAs due to having identical protospacers. **(B)** Summary of editing outcomes in transgenic T0 lines (i.e., monoallelic and non-chimeric) by different CGBEs in poplar **(C)** Base-edited T0 lines (i.e., monoallelic and non-chimeric). at the PtPDS-sgRNA8 site with editing frequencies quantified by NGS and Hi-Tom analysis.

We also wanted to assess the CGBEs in a dicot plant species using stable transformation. We chose a Populus hybrid (Populus tremula × P. alba hybrid clone INRA 717-1B4) in which efficient C-to-T and A-to-G base editing was recently demonstrated (Li et al., 2021a). Two sgRNAs with canonical NGG PAMs were designed, with sgRNA8 targeting *PtPDS1* and *PtPDS2*, and with sgRNA9 targeting *PtPDS1*. In all cases, both *P. alba* and *P. tremula* genomes were targeted due to the presence of identical target sequences (Figure 5A). All three CGBE fusion proteins (pYPQ265K, pYPQ265N1, and pYPQ265O1) were expressed under an Arabidopsis Ubiquitin 10 (AtUbi10) promoter and the sgRNAs were expressed under an AtU3 promoter. For each construct, 32 T0 lines were generated and analyzed with the Hi-Tom NGS platform (Liu et al., 2019). Interestingly, among all 192 T0 lines assessed, only four lines contained base edits and they were all derived from the pYPQ265K CGBE with sgRNA8 (Figure 5B). Among them, two lines (4023-7 and 4023-22) contained C-to-G editing at sixth and eighth positions, respectively (Figure 5C). The two other lines (4023-4 and 4023-25) contained C-to-T editing at the sixth and seventh positions, respectively (Figure 5C). Based on the percentages of NGS reads, the 023-22 line was a monoallelic line with C8-to-G8 base change (Figure 5C). Interestingly, although sgRNA8 could also target PtPDS2 (Figure 5A), no base edits could be found in this gene, suggesting PtPDS1 was more accessible than PtPDS2 for base editing in this poplar hybrid. Furthermore, no indels were found among all the T0 lines analyzed. Taken together, the data suggest the UNG-rAPOBEC1 (R333A)-based CGBE (pYPQ265K) can generated C-to-G editing with undetectable indel byproduct formation in poplar.

## Discussion

Despite the great progress in achieving highly efficient C-to-T and A-to-G base transition editing in plants, plant transversion editors have not been previously reported. Here we compared three CGBEs toward targeted C-to-G editing in plants. Our assessment in rice and tomato protoplasts showed that these CGBEs, not the BE3, could induce C-to-G editing at the target sites. pYPQ265K, which is based on UNG-rAPOBEC1 (R33A), appeared to be the best among the three CGBEs for generating the overall higher C-to-G conversion rates. However, C-to-T edits still predominated among the editing outcomes, suggesting room for improvement in achieving high C-to-G base editing purity by minimizing byproduct formation. Consistent with reports in human cell lines (Chen et al., 2021; Kurt et al., 2021; Zhao et al., 2021), these three CGBEs all greatly improved the ratios of C-to-G editing over C-to-T editing, as the control BE3 barely generated any C-to-G editing events in rice protoplasts (Figure 1) and tomato protoplasts (Figure 2). Such effects could be partly explained by the removal of UGI and addition of UNG or rXRCC1 (Chen et al., 2021; Kurt et al., 2021; Zhao et al., 2021). Evaluation of editing windows for these three CGBEs in rice and tomato protoplasts showed editing preference for C6 in the 20-nucleotide target sequence, which is a general feature reported for CGBEs (Chen et al., 2021; Kurt et al., 2021; Zhao et al., 2021). rAPOBEC1 used in these CGBEs are known to have poor editing activity at GC context when a targeting C is proceeded by a G (Komor et al., 2016). By contrast, when the target C is flanked by A and/or T, it is highly likely to be edited by CGBEs, according to data in human cells (Kurt et al., 2021). Interestingly, all the C-to-G edited stable lines in rice and poplar seemed to obey this rule, showing editing in the TC, AC, and CC context (Figures 4, 5). In addition, these CGBEs induced very low levels of C-to-A transversion editing in rice and tomato protoplasts (Supplementary Figures S2, S6), consistent with the observations in human cells (Chen et al., 2021; Kurt et al., 2021; Zhao et al., 2021).

While these general rules seem to hold true in human and plant cells, we also discovered major differences for the CGBEs in plants compared to in human cells. First, the overall C-to-G editing frequencies in rice and tomato cells (0.4–1.8%) were nearly one magnitude lower than those reported in human cells (Chen et al., 2021; Kurt et al., 2021; Zhao et al., 2021). Furthermore, these CGBEs still produced much more C-to-T editing events than C-to-G editing events in the protoplasts of rice and tomato, as well as in stable transgenic rice lines. While this could be partly explained by protoplasts cells in our experiments being mostly non-dividing, our data in stably transformed rice and poplar plants also showed overall low C-to-G editing frequencies. In rice, only the rXRCC1-based CGBEs (pYPQ265O1 and pYPQ265O2) generated pure C-to-G editing events (Figure 4). In poplar, only the UNG-rAPOBEC1 (R33A)-based CGBE (pYPQ265K) produced pure C-to-G editing events (Figure 5). Second, although these CGBEs all generated relatively high levels of indel frequencies in the protoplasts (Supplementary Figures S3, S5), indel mutations were undetectable for most CGBE constructs in stably transformed plants (Figures 4, 5). These observations suggest that the performance of CGBEs is highly dependent on the cell cycles and DNA repair pathways in plants.

To expand the targeting ranges, we developed CGBEs based on PAM-less SpRY (Walton et al., 2020; Ren et al., 2021b). These SpRY CGBEs were able to edit PAM-relaxed target sites, albeit with low efficiency in rice protoplasts (Figure 3), which could be partly due to vector self-editing, a feature of PAM-less SpRY systems (Ren et al., 2021b). Remarkably, one SpRY CGBE, pYPQ265O2, generated 38.0% C-to-G editing (8 out of 21 lines) at the OsALS-sgRNA22 site in the T0 lines (Figures 4A,C). Interestingly, C-to-G editing by the same construct only generated 0.1% frequency in rice protoplasts (Figure 3B). Interestingly, the UNG-rAPOBEC1 (R33A)-based pYPQ265K generated equivalent C-to-G editing frequency to C-to-T editing frequency (6.25 vs. 6.25% at one target site in poplar (Figure 5B). It is of note that germline transmission of these observed C-to-G editing events need to be further investigated, especially in rice. The discrepancy for C-to-G editing frequencies and outcomes between protoplasts and stable plants further supports that differential DNA repair activities in different cell types and plant species play an important role in the base editing process. Therefore, it would be very important to understand DNA repair, especially the BER pathway, in different plant species, tissue types, and at different cell cycle stages. We envision that harnessing plant-sourced BER pathway genes, in a similar approach to the development of rXRCC1-based CGBE (Chen et al., 2021), may aid the future development of CGBEs with improved C-to-G base editing efficiency in plants.

Here, we closely compared three CGBE platforms, which are top-performing CGBEs in human cells (Chen et al., 2021; Kurt et al., 2021; Zhao et al., 2021), hoping to identify the best performer for C-to-G base editing in plants. However, our data do not indicate there is a clear winner among the tested CGBEs. For example, the UNG-rAPOBEC1 (R33A)-based pYPQ265K appeared to have the highest C-to-G editing frequencies in rice and tomato protoplasts at the canonical NGG PAMs (Figures 1, 2). However, it is the rXRCC1-based pYPQ265O1 and pYPQ265O2 that generated pure C-to-G base editing lines in rice (Figure 4). Though, pYPQ265K was successful in producing pure C-to-G base editing lines in poplar (Figure 5). When we compared the three CGBE platforms with PAM-less SpRY, the rAPOBEC1-nSpRY-UNG (pYPQ265N2) appeared to be very robust, editing six out of nine target sites in rice protoplasts (Figure 3B), suggesting possible differential compatibility of these CGBE systems with the Cas protein. Yet, pYPQ265N2 did not generate stably edited lines in rice. Furthermore, the fact that SpRY-based pYPQ265O2 could generate 38.0% C-to-G editing frequency at one target site in rice suggests there is potentially a strong context dependency for editing outcomes. It might be possible to resolve the mechanism through mining a large editing data set. While we were preparing this manuscript, a recent study reported a similar phenomenon in human cells (Koblan et al., 2021). The authors only observed moderately improved C-to-G editing efficiency after replacing the *E. coli* UNG with a UNG ortholog from *Mycobacterium smegmatis* (UdgX). After establishing an APO-UdgX-Cas9n (AXC) CGBE platform, the authors used CRISPRi to screen a library of 476 DNA repair genes to uncover determinants of base editing outcomes in human cells. The resulting gene candidates were then used for enhancing C-to-G editing as protein fusions. Interestingly, no single CGBE outperformed other CGBEs at all target sites, echoing our findings in plants. The authors ended up using machine learning to develop a program termed CGBE-Hive for predicting the performance of individual CGBEs based on a large amount of editing data generated in human cells (Koblan et al., 2021). Thus, it is envisioned that a similar approach in plants may be needed for understanding the editing preference of CGBEs in plants to advance the use of C-to-G editing and improve reliability to aid basic and applied plant research. With more advances in guide RNA library based CRISPR screens in plants, it could be realized in the future.

## Conclusion

In this study, we assessed a total of six CGBEs for editing NGG PAM sites as well as PAM-less target sites in plants. Albeit low efficiencies, C-to-G editing was achieved in stable transformed lines of rice and poplar. This work represents a first step toward achieving efficient C-to-G base editing in plants. Future research is warranted for the development of improved CGBEs with high editing activity and purity in plants.

## Data Availability

The datasets presented in this study can be found in online repositories. The names of the repository/repositories and accession number(s) can be found below: https://www.ncbi.nlm.nih.gov/, PRJNA747640 https://www.ncbi.nlm.nih.gov/, PRJNA747683.
