# Supplementary material for: Exploring C-To-G Base Editing in Rice, Tomato, and Poplar
Source: Front Genome Ed. 2021 Sep 15;3:756766. doi: 10.3389/fgeed.2021.756766 (PMC8525388; doi:10.3389/fgeed.2021.756766)
Supplement: Supplementary file 1 [file Table1.DOCX]

**Supplementary Table 1. Oligos and gBlocks used in this study**

| **Name** | **Sequence 5'-3'** | **Description** |
| --- | --- | --- |
| OsALS-sgR32-F | tggcaCAGGTCCCCCGCCGCATGAT | Forward oligo to prepare sgRNA OsALS-sgRNA32 |
| OsALS-sgR32-R | aaacATCATGCGGCGGGGGACCTGt | Reverse oligo to prepare sgRNA OsALS-sgRNA32 |
| C-Rich5-F | tgtgGCGCGCCTCGATGATACATCC | Forward oligo to prepare sgRNA OsCGRS55-sgRNA-sgRNA |
| C-Rich5-R | aaacGGATGTATCATCGAGGCGCGC | Reverse oligo to prepare sgRNA OsCGRS55-sgRNA-sgRNA |
| SAgo7-CtoG-gR1-F | ggtcaCACCACAACAGATTCTGTGG | Forward oligo to prepare sgRNA SAgo7-gR1 |
| SAgo7-CtoG-gR1-R | aaacCCACAGAATCTGTTGTGGTGt | Reverse oligo to prepare sgRNA SAgo7-gR1 |
| SAgo7-CtoG-gR2-F | ggtcAAATGCAAGGGTTCAAACTG | Forward oligo to prepare sgRNA SAgo7-gR2 |
| SAgo7-CtoG-gR2-R | aaacCAGTTTGAACCCTTGCATTT | Reverse oligo to prepare sgRNA SAgo7-gR2 |
| SAgo7-CtoG-gR3-F | ggtcaTCAGCCTCCTAAGCTTAAACT | Forward oligo to prepare sgRNA SAgo7-gR3 |
| SAgo7-CtoG-gR3-R | aaacAGTTTAAGCTTAGGAGGCTGAt | Reverse oligo to prepare sgRNA SAgo7-gR3 |
| SAgo7-CtoG-gR4-F | gattgAAGATCTCAGCGCGATGGTT | Forward oligo to prepare sgRNA SAgo7-gR4 |
| SAgo7-CtoG-gR4-R | aaacAACCATCGCGCTGAGATCTTc | Reverse oligo to prepare sgRNA SAgo7-gR4 |
| OsEPSPS-sgR31-F | TGGCAcagttccagcgttccccaag | Forward oligo to prepare sgRNA OsEPSPS-sgRNA31 |
| OsEPSPS-sgR31-R | AAACcttggggaacgctggaactgT | Reverse oligo to prepare sgRNA OsEPSPS-sgRNA31 |
| OsALS-sgR24-F | gtgtGCCCCCACTTGGGATCATAG | Forward oligo to prepare sgRNA OsALS-sgRNA24 |
| OsALS-sgR24-R | aaacCTATGATCCCAAGTGGGGGC | Reverse oligo to prepare sgRNA OsALS-sgRNA24 |
| OsEPSPS-sgR30-F | TGGCagttccagcgttccccaaga | Forward oligo to prepare sgRNA OsEPSPS-sgRNA30 |
| OsEPSPS-sgR30-R | AAACtcttggggaacgctggaact | Reverse oligo to prepare sgRNA OsEPSPS-sgRNA30 |
| OsALS-sgR147-F | GTGTggacgcgccgcccgggtagg | Forward oligo to prepare sgRNA OsALS-sgRNA147 |
| OsALS-sgR147-R | AAACcctacccgggcggcgcgtcc | Reverse oligo to prepare sgRNA OsALS-sgRNA147 |
| OsALS-sgR22-F | tggcaCCCCACTTGGGATCATAGGC | Forward oligo to prepare sgRNA OsALS-sgRNA22 |
| OsALS-sgR22-R | aaacGCCTATGATCCCAAGTGGGGt | Reverse oligo to prepare sgRNA OsALS-sgRNA22 |
| OsALS-sgR31-F | tggcAGGTCCCCCGCCGCATGATC | Forward oligo to prepare sgRNA OsALS-sgRNA31 |
| OsALS-sgR31-R | aaacGATCATGCGGCGGGGGACCT | Reverse oligo to prepare sgRNA OsALS-sgRNA31 |
| OsALS-sgR150-F | GTGTgccttccaggagacgcccat | Forward oligo to prepare sgRNA OsALS-sgRNA150 |
| OsALS-sgR150-R | AAACatgggcgtctcctggaaggc | Reverse oligo to prepare sgRNA OsALS-sgRNA150 |
| OsALS-sgR32-F | tggcaCAGGTCCCCCGCCGCATGAT | Forward oligo to prepare sgRNA OsALS-sgRNA32 |
| OsALS-sgR32-R | aaacATCATGCGGCGGGGGACCTGt | Reverse oligo to prepare sgRNA OsALS-sgRNA32 |
| PtPDS-sgRNA8-F | ggtcACGCACCCGCAAAGGAGGG | Forward oligo to prepare sgRNA PDS1-sgRNA08 |
| PtPDS-sgRNA8-R | aaacCCCTCCTTTGCGGGTGCGT | Reverse oligo to prepare sgRNA PDS1-sgRNA08 |
| PtPDS-sgRNA9-F | ggtcaGACTACACAAAACAAAAGTACT | Forward oligo to prepare sgRNA PDS1-sgRNA09 |
| PtPDS-sgRNA9-R | aaacAGTACTTTTGTTTTGTGTAGTCt | Reverse oligo to prepare sgRNA PDS1-sgRNA09 |
| NGS-SAgo7-1-F1 | ACAGTGACAGCCTCAAAGGTGCTCCA | Forward oligo to amplify the SAgo7-gR1 target site for NGS |
| NGS-SAgo7-1-R1 | CAACTAGATTAGCCTAGCAATATCCT | Reverse oligo to amplify the SAgo7-gR1 target site for NGS |
| NGS-SAgo7-1-F2t | AGTCAAACAGCCTCAAAGGTGCTCCA | Forward oligo to amplify the SAgo7-gR1 target site for NGS |
| NGS-SAgo7-1-R2 | CACGATGATTAGCCTAGCAATATCCT | Reverse oligo to amplify the SAgo7-gR1 target site for NGS |
| NGS-SAgo7-2-F1 | AGTCAACTGGACAGCCATGTCTTCGA | Forward oligo to amplify the SAgo7-gR2 target site for NGS |
| NGS-SAgo7-2-R1 | CACGATTCCCGATGCTTGTCTCAGCG | Reverse oligo to amplify the SAgo7-gR2 target site for NGS |
| NGS-SAgo7-2-F2 | AGTTCCCTGGACAGCCATGTCTTCGA | Forward oligo to amplify the SAgo7-gR2 target site for NGS |
| NGS-SAgo7-2-R2 | CCAACATCCCGATGCTTGTCTCAGCG | Reverse oligo to amplify the SAgo7-gR2 target site for NGS |
| NGS-SAgo7-3-F1 | AGTTCCGTGGCAATCATGCGAGTGAC | Forward oligo to amplify the SAgo7-gR3 target site for NGS |
| NGS-SAgo7-3-R1 | CCAACATCGAAGACATGGCTGTCCAG | Reverse oligo to amplify the SAgo7-gR3 target site for NGS |
| NGS-SAgo7-3-F2 | TTAGGCGTGGCAATCATGCGAGTGAC | Forward oligo to amplify the SAgo7-gR3 target site for NGS |
| NGS-SAgo7-3-R2 | CACTCATCGAAGACATGGCTGTCCAG | Reverse oligo to amplify the SAgo7-gR3 target site for NGS |
| NGS-SAgo7-4-F1 | TAGCTTTCCGTCGCTGCTGTAGTTG | Forward oligo to amplify the SAgo7-gR4 target site for NGS |
| NGS-SAgo7-4-R1 | CAAAAGCAGCCGAATTGCTTGGAGC | Reverse oligo to amplify the SAgo7-gR4 target site for NGS |
| NGS-SAgo7-4-F2 | CTTGTATCCGTCGCTGCTGTAGTTG | Forward oligo to amplify the SAgo7-gR4 target site for NGS |
| NGS-SAgo7-4-R2 | TATAATCAGCCGAATTGCTTGGAGC | Reverse oligo to amplify the SAgo7-gR4 target site for NGS |
| PtPDS-HiTom-F2 | ggagtgagtacggtgtgcTTGGAAATTCTTCTGCTAAA | Forward oligo to amplify PDS1-sgRNA8 for Hi-TOM NGS |
| PtPDS-HiTom-R2 | gagttggatgctggatggCTAAGAAATTCACCGTGTTA | Reverse oligo to amplify PDS1-sgRNA8 for Hi-TOM NGS |
| PtPDS-HiTom-F3 | ggagtgagtacggtgtgcCTTGCAAAGATCTCCGATAG | Forward oligo to amplify PDS1-sgRNA9 for Hi-TOM NGS |
| PtPDS-HiTom-R3 | gagttggatgctggatggCTAGATCAGACAGTGAGTAC | Reverse oligo to amplify PDS1-sgRNA9 for Hi-TOM NGS |
| Hi-TOM-F-1 | ACACTCTTTCCCTACACGACGCTCTTCCGATCTgcttGCGTtggagtgagtacggtgtgc | Forward barcoding oligo for Hi-TOM NGS |
| Hi-TOM-F-2 | ACACTCTTTCCCTACACGACGCTCTTCCGATCTgcttGTAGtggagtgagtacggtgtgc | Forward barcoding oligo for Hi-TOM NGS |
| Hi-TOM-F-3 | ACACTCTTTCCCTACACGACGCTCTTCCGATCTgcttACGCtggagtgagtacggtgtgc | Forward barcoding oligo for Hi-TOM NGS |
| Hi-TOM-F-4 | ACACTCTTTCCCTACACGACGCTCTTCCGATCTgcttCTCGtggagtgagtacggtgtgc | Forward barcoding oligo for Hi-TOM NGS |
| Hi-TOM-F-5 | ACACTCTTTCCCTACACGACGCTCTTCCGATCTgcttGCTCtggagtgagtacggtgtgc | Forward barcoding oligo for Hi-TOM NGS |
| Hi-TOM-F-6 | ACACTCTTTCCCTACACGACGCTCTTCCGATCTgcttAGTCtggagtgagtacggtgtgc | Forward barcoding oligo for Hi-TOM NGS |
| Hi-TOM-F-7 | ACACTCTTTCCCTACACGACGCTCTTCCGATCTgcttCGACtggagtgagtacggtgtgc | Forward barcoding oligo for Hi-TOM NGS |
| Hi-TOM-F-8 | ACACTCTTTCCCTACACGACGCTCTTCCGATCTgcttGATGtggagtgagtacggtgtgc | Forward barcoding oligo for Hi-TOM NGS |
| Hi-TOM-F-9 | ACACTCTTTCCCTACACGACGCTCTTCCGATCTgcttATACtggagtgagtacggtgtgc | Forward barcoding oligo for Hi-TOM NGS |
| Hi-TOM-F-10 | ACACTCTTTCCCTACACGACGCTCTTCCGATCTgcttCACAtggagtgagtacggtgtgc | Forward barcoding oligo for Hi-TOM NGS |
| Hi-TOM-F-11 | ACACTCTTTCCCTACACGACGCTCTTCCGATCTgcttGTGCtggagtgagtacggtgtgc | Forward barcoding oligo for Hi-TOM NGS |
| Hi-TOM-F-12 | ACACTCTTTCCCTACACGACGCTCTTCCGATCTgcttACTAtggagtgagtacggtgtgc | Forward barcoding oligo for Hi-TOM NGS |
| Hi-TOM-R-A | GACTGGAGTTCAGACGTGTGCTCTTCCGATCTctgtGCGTtgagttggatgctggatgg | Reverse barcoding oligo for Hi-TOM NGS |
| Hi-TOM-R-B | GACTGGAGTTCAGACGTGTGCTCTTCCGATCTctgtGTAGtgagttggatgctggatgg | Reverse barcoding oligo for Hi-TOM NGS |
| Hi-TOM-R-C | GACTGGAGTTCAGACGTGTGCTCTTCCGATCTctgtACGCtgagttggatgctggatgg | Reverse barcoding oligo for Hi-TOM NGS |
| Hi-TOM-R-D | GACTGGAGTTCAGACGTGTGCTCTTCCGATCTctgtCTCGtgagttggatgctggatgg | Reverse barcoding oligo for Hi-TOM NGS |
| Hi-TOM-R-E | GACTGGAGTTCAGACGTGTGCTCTTCCGATCTctgtGCTCtgagttggatgctggatgg | Reverse barcoding oligo for Hi-TOM NGS |
| Hi-TOM-R-F | GACTGGAGTTCAGACGTGTGCTCTTCCGATCTctgtAGTCtgagttggatgctggatgg | Reverse barcoding oligo for Hi-TOM NGS |
| Hi-TOM-R-G | GACTGGAGTTCAGACGTGTGCTCTTCCGATCTctgtCGACtgagttggatgctggatgg | Reverse barcoding oligo for Hi-TOM NGS |
| Hi-TOM-R-H | GACTGGAGTTCAGACGTGTGCTCTTCCGATCTctgtGATGtgagttggatgctggatgg | Reverse barcoding oligo for Hi-TOM NGS |
| OsALS-32-F1 | GGTAGCtcgccgacgcgctgctcgac | forward oligo to amplify the OsALS-sgRNA32 target site for NGS |
| OsALS-32-R1 | CATGGCggaagaaggcttcctgtatgacgc | reverse oligo to amplify the OsALS-sgRNA32 target site for NGS |
| 3741-HTS-F1 | ATCACGtcgccgacgcgctgctcgac | forward oligo to amplify the OsALS-sgRNA32 target site for NGS |
| 3741-HTS-R1 | CAAAAGggaagaaggcttcctgtatgacgc | reverse oligo to amplify the OsALS-sgRNA32 target site for NGS |
| 3741-HTS-F2 | ACAGTGtcgccgacgcgctgctcgac | forward oligo to amplify the OsALS-sgRNA32 target site for NGS |
| 3741-HTS-R2 | CAACTAggaagaaggcttcctgtatgacgc | reverse oligo to amplify the OsALS-sgRNA32 target site for NGS |
| 3741-HTS-F3 | ACTTGAtcgccgacgcgctgctcgac | forward oligo to amplify the OsALS-sgRNA32 target site for NGS |
| 3741-HTS-R3 | CACCGGggaagaaggcttcctgtatgacgc | reverse oligo to amplify the OsALS-sgRNA32 target site for NGS |
| 3795-HTS-F1 | TTAGGCtcgccgacgcgctgctcgac | forward oligo to amplify the OsALS-sgRNA32 target site for NGS |
| 3795-HTS-R1 | CATTTTggaagaaggcttcctgtatgacgc | reverse oligo to amplify the OsALS-sgRNA32 target site for NGS |
| 3795-HTS-F2 | TGACCAtcgccgacgcgctgctcgac | forward oligo to amplify the OsALS-sgRNA32 target site for NGS |
| 3795-HTS-R2 | CCAACAggaagaaggcttcctgtatgacgc | reverse oligo to amplify the OsALS-sgRNA32 target site for NGS |
| 3795-HTS-F3 | TAGCTTtcgccgacgcgctgctcgac | forward oligo to amplify the OsALS-sgRNA32 target site for NGS |
| 3795-HTS-R3 | CGGAATggaagaaggcttcctgtatgacgc | reverse oligo to amplify the OsALS-sgRNA32 target site for NGS |
| Q55-F1 | GGCTACAGTGCATGCAGCTAGCTAGGT | forward oligo to amplify the OsCGRS55-sgRNA target site for NGS |
| Q55-R1 | ATTCCTCGAGCTGCTGTGCTTCCTGT | reverse oligo to amplify the OsCGRS55-sgRNA target site for NGS |
| 3742-HTS-F1 | CGATGTAGTGCATGCAGCTAGCTAGGT | forward oligo to amplify the OsCGRS55-sgRNA target site for NGS |
| 3742-HTS-RN1 | ACTGATCGAGCTGCTGTGCTTCCTGT | reverse oligo to amplify the OsCGRS55-sgRNA target site for NGS |
| 3742-HTS-F2 | GTGAAAAGTGCATGCAGCTAGCTAGGT | forward oligo to amplify the OsCGRS55-sgRNA target site for NGS |
| 3742-HTS-R2 | TACAGCCGAGCTGCTGTGCTTCCTGT | reverse oligo to amplify the OsCGRS55-sgRNA target site for NGS |
| 3742-HTS-F3 | CTTGTAAGTGCATGCAGCTAGCTAGGT | forward oligo to amplify the OsCGRS55-sgRNA target site for NGS |
| 3742-HTS-R3 | ATTCCTCGAGCTGCTGTGCTTCCTGT | reverse oligo to amplify the OsCGRS55-sgRNA target site for NGS |
| 3748-HTS-F1 | GGTAGCAGTGCATGCAGCTAGCTAGGT | forward oligo to amplify the OsCGRS55-sgRNA target site for NGS |
| 3748-HTS-R1 | TATAATCGAGCTGCTGTGCTTCCTGT | reverse oligo to amplify the OsCGRS55-sgRNA target site for NGS |
| 3748-HTS-F2 | GTCCGCAGTGCATGCAGCTAGCTAGGT | forward oligo to amplify the OsCGRS55-sgRNA target site for NGS |
| 3748-HTS-R2 | CACTCACGAGCTGCTGTGCTTCCTGT | reverse oligo to amplify the OsCGRS55-sgRNA target site for NGS |
| 3748-HTS-F3 | GTGAAAAGTGCATGCAGCTAGCTAGGT | forward oligo to amplify the OsCGRS55-sgRNA target site for NGS |
| 3748-HTS-R3 | CAGGCGCGAGCTGCTGTGCTTCCTGT | reverse oligo to amplify the OsCGRS55-sgRNA target site for NGS |
| 3794-HTS-F1 | CGATGTgaagtccgtgccgccatca | forward oligo to amplify the OsALS-sgRNA24 target site for NGS |
| 3794-HTS-R1 | ACTGATggtcattcaggtcaaacataggcc | reverse oligo to amplify the OsALS-sgRNA24 target site for NGS |
| 3794-HTS-F2 | GTGAAAgaagtccgtgccgccatca | forward oligo to amplify the OsALS-sgRNA24 target site for NGS |
| 3794-HTS-R2 | TACAGCggtcattcaggtcaaacataggcc | reverse oligo to amplify the OsALS-sgRNA24 target site for NGS |
| 3794-HTS-F3 | CTTGTAgaagtccgtgccgccatca | forward oligo to amplify the OsALS-sgRNA24 target site for NGS |
| 3794-HTS-R3 | ATTCCTggtcattcaggtcaaacataggcc | reverse oligo to amplify the OsALS-sgRNA24 target site for NGS |
| OsALS-24-F | AGTCAAgaagtccgtgccgccatca | forward oligo to amplify the OsALS-sgRNA24 target site for NGS |
| OsALS-24-R | CTATACggtcattcaggtcaaacataggcc | reverse oligo to amplify the OsALS-sgRNA24 target site for NGS |
| 3793-HTS-F1 | ATCACGgaagtccgtgccgccatca | forward oligo to amplify the OsALS-sgRNA22 target site for NGS |
| 3793-HTS-R1 | CAAAAGggtcattcaggtcaaacataggcc | reverse oligo to amplify the OsALS-sgRNA22 target site for NGS |
| 3793-HTS-F2 | ACAGTGgaagtccgtgccgccatca | forward oligo to amplify the OsALS-sgRNA22 target site for NGS |
| 3793-HTS-R2 | CAACTAggtcattcaggtcaaacataggcc | reverseoligo to amplify the OsALS-sgRNA22 target site for NGS |
| 3793-HTS-F3 | ACTTGAgaagtccgtgccgccatca | forward oligo to amplify the OsALS-sgRNA22 target site for NGS |
| 3793-HTS-R3 | CACCGGggtcattcaggtcaaacataggcc | reverseoligo to amplify the OsALS-sgRNA22 target site for NGS |
| OsALS-22-F | ATGTCAgaagtccgtgccgccatca | forward oligo to amplify the OsALS-sgRNA22 target site for NGS |
| OsALS-22-R | CTCAGAggtcattcaggtcaaacataggcc | reverse oligo to amplify the OsALS-sgRNA22 target site for NGS |
| 3798-HTS-F1 | TGACCAgagccccgcaagggcgcgga | forward oligo to amplify the OsALS-sgRNA147 target site for NGS |
| 3798-HTS-R1 | CTATACcgtacccggacgccgcgaac | reverse oligo to amplify the OsALS-sgRNA147 target site for NGS |
| 3798-HTS-F2 | TAGCTTgagccccgcaagggcgcgga | forward oligo to amplify the OsALS-sgRNA147 target site for NGS |
| 3798-HTS-R2 | CTCAGAcgtacccggacgccgcgaac | reverse oligo to amplify the OsALS-sgRNA147 target site for NGS |
| 3798-HTS-F3 | AGTTCCgagccccgcaagggcgcgga | forward oligo to amplify the OsALS-sgRNA147 target site for NGS |
| 3798-HTS-R3 | CTATACcgtacccggacgccgcgaac | reverse oligo to amplify the OsALS-sgRNA147 target site for NGS |
| OsALS-147-F | CAGATCgagccccgcaagggcgcgga | forward oligo to amplify the OsALS-sgRNA147 target site for NGS |
| OsALS-147-R | TCGGCAcgtacccggacgccgcgaac | reverse oligo to amplify the OsALS-sgRNA147 target site for NGS |
| 3796-HTS-F1 | GTAGAGtcgccgacgcgctgctcgac | forward oligo to amplify the OsALS-sgRNA31 target site for NGS |
| 3796-HTS-R1 | CGGAATggaagaaggcttcctgtatgacgc | reverse oligo to amplify the OsALS-sgRNA31 target site for NGS |
| 3796-HTS-F2 | GTCCGCtcgccgacgcgctgctcgac | forward oligo to amplify the OsALS-sgRNA31 target site for NGS |
| 3796-HTS-R2 | CTAGCTggaagaaggcttcctgtatgacgc | reverse oligo to amplify the OsALS-sgRNA31 target site for NGS |
| 3796-HTS-F3 | GTAGAGtcgccgacgcgctgctcgac | forward oligo to amplify the OsALS-sgRNA31 target site for NGS |
| 3796-HTS-R3 | CAAAAGggaagaaggcttcctgtatgacgc | reverse oligo to amplify the OsALS-sgRNA31 target site for NGS |
| OsALS-31-F | TGACCAtcgccgacgcgctgctcgac | forward oligo to amplify the OsALS-sgRNA31 target site for NGS |
| OsALS-31-R | CAGGCGggaagaaggcttcctgtatgacgc | reverse oligo to amplify the OsALS-sgRNA31 target site for NGS |
| 3800-HTS-F1 | GTTTCGtcgccgacgcgctgctcgac | forward oligo to amplify the OsALS-sgRNA150 target site for NGS |
| 3800-HTS-R1 | TAATCGggaagaaggcttcctgtatgacgc | reverse oligo to amplify the OsALS-sgRNA150 target site for NGS |
| 3800-HTS-F2 | GCCAATtcgccgacgcgctgctcgac | forward oligo to amplify the OsALS-sgRNA150 target site for NGS |
| 3800-HTS-R2 | TACAGCggaagaaggcttcctgtatgacgc | reverse oligo to amplify the OsALS-sgRNA150 target site for NGS |
| 3800-HTS-F3 | ACTTGAtcgccgacgcgctgctcgac | forward oligo to amplify the OsALS-sgRNA150 target site for NGS |
| 3800-HTS-R3 | TAATCGggaagaaggcttcctgtatgacgc | reverse oligo to amplify the OsALS-sgRNA150 target site for NGS |
| OsALS-150-F | GTGGCCtcgccgacgcgctgctcgac | forward oligo to amplify the OsALS-sgRNA150 target site for NGS |
| OsALS-150-R | TCGAAGggaagaaggcttcctgtatgacgc | reverse oligo to amplify the OsALS-sgRNA150 target site for NGS |
| 3807-HTS-F1 | GTAGAGgctgtagtcgttggctgtgg | forward oligo to amplify the OsEPSPS-sgRNA31 target site for NGS |
| 3807-HTS-R1 | CGGAATcataccccatgaattccatacATA | reverse oligo to amplify the OsEPSPS-sgRNA31 target site for NGS |
| 3807-HTS-F2 | GTCCGCgctgtagtcgttggctgtgg | forward oligo to amplify the OsEPSPS-sgRNA31 target site for NGS |
| 3807-HTS-R2 | CTAGCTcataccccatgaattccatacATA | reverse oligo to amplify the OsEPSPS-sgRNA31 target site for NGS |
| 3807-HTS-F3 | GTAGAGgctgtagtcgttggctgtgg | forward oligo to amplify the OsEPSPS-sgRNA31 target site for NGS |
| 3807-HTS-R3 | CAAAAGcataccccatgaattccatacATA | reverse oligo to amplify the OsEPSPS-sgRNA31 target site for NGS |
| OsEPSPE-31-F | GTGGCCgctgtagtcgttggctgtgg | forward oligo to amplify the OsEPSPS-sgRNA31 target site for NGS |
| OsEPSPE-31-R | TATAATcataccccatgaattccatacATA | reverse oligo to amplify the OsEPSPS-sgRNA31 target site for NGS |
| 3808-HTS-F1 | GATCAGgctgtagtcgttggctgtgg | forward oligo to amplify the OsEPSPS-sgRNA30 target site for NGS |
| 3808-HTS-R1 | CATTTTcataccccatgaattccatacATA | reverse oligo to amplify the OsEPSPS-sgRNA30 target site for NGS |
| 3808-HTS-F2 | GTGAAAgctgtagtcgttggctgtgg | forward oligo to amplify the OsEPSPS-sgRNA30 target site for NGS |
| 3808-HTS-R2 | CTATACcataccccatgaattccatacATA | reverse oligo to amplify the OsEPSPS-sgRNA30 target site for NGS |
| 3808-HTS-F3 | TTAGGCgctgtagtcgttggctgtgg | forward oligo to amplify the OsEPSPS-sgRNA30 target site for NGS |
| 3808-HTS-R3 | CTAGCTcataccccatgaattccatacATA | reverse oligo to amplify the OsEPSPS-sgRNA30 target site for NGS |
| OsEPSPE-30-F | GTGGCCgctgtagtcgttggctgtgg | forward oligo to amplify the OsEPSPS-sgRNA30 target site for NGS |
| OsEPSPE-30-R | TCGAAGcataccccatgaattccatacATA | reverse oligo to amplify the OsEPSPS-sgRNA30 target site for NGS |
| 266E-INS_fwd | ctcgatcggcCTCGcTATTGGGACTAACTCTG | Forward oligo for preparing Gateway compatible attL1-attR5 entry clones |
| 266E-INS_rev | gcgtctcgtaGAGGCCGGTAATCGACTG | Reverse oligo for preparing Gateway compatible attL1-attR5 entry clones |
| 266E-BB_fwd | taccggcctcTACGAGACGCGCATCGAC | Forward oligo for preparing Gateway compatible attL1-attR5 entry clones |
| 266E-BB_rev | caataGcgagGCCGATCGAGTACTTCTTGTC | Reverse oligo for preparing Gateway compatible attL1-attR5 entry clones |
| 265N1-BB_F | gcccattgactggaaggagctgTCAGGTGGCTCCCCAAAGAAG | Forward oligo for preparing Gateway compatible attL1-attR5 entry clones |
| 265N1-BB_R | tcttccagctctctccaaacatTGACCCGCCTGATCCGTCTC | Reverse oligo for preparing Gateway compatible attL1-attR5 entry clones |
| 265O1-BB_F | ttacggcgttgttccgcaggcgTCAGGTGGCTCCCCAAAGAAG | Forward oligo for preparing Gateway compatible attL1-attR5 entry clones |
| 265O1-BB_R | catggcgaaggctgatttcaggTGACCCGCCTGATCCGTCTC | Reverse oligo for preparing Gateway compatible attL1-attR5 entry clones |
| Attl1-APB-F | ccagtcttaagctcgggccccaa | Oligo for Sanger sequencing of Gateway compatible attL1-attR5 entry clones |
| 165-F3 | gctacctgcaggagattttc | Oligo for Sanger sequencing of Gateway compatible attL1-attR5 entry clones |
| 165-F4 | acgccatcctcctgtctga | Oligo for Sanger sequencing of Gateway compatible attL1-attR5 entry clones |
| 165-F5 | tctacaacgagctgactaaggt | Oligo for Sanger sequencing of Gateway compatible attL1-attR5 entry clones |
| 165-F6 | gaaggtcatgggcaggcacaa | Oligo for Sanger sequencing of Gateway compatible attL1-attR5 entry clones |
| 165-F7 | cgggagatcaacaattaccaccat | Oligo for Sanger sequencing of Gateway compatible attL1-attR5 entry clones |
| 165-F8 | ctggaggcgaagggctacaa | Oligo for Sanger sequencing of Gateway compatible attL1-attR5 entry clones |
| 165-F10 | GTCCTCTTGATTACGGATAC | Oligo for Sanger sequencing of Gateway compatible attL1-attR5 entry clones |
| zCas9-R1 | ttgaacttctttgagggcacc | Oligo for Sanger sequencing of Gateway compatible attL1-attR5 entry clones |
| zCas9-R2 | CGT ACA CGA ACT CGC TCT C | Oligo for Sanger sequencing of Gateway compatible attL1-attR5 entry clones |
| zCas9-F11 | gtatgtggaccaggagctg | Oligo for Sanger sequencing of Gateway compatible attL1-attR5 entry clones |
| Nos-Term-R2 | aatcatcgcaagaccggcaacagg | Oligo for Sanger sequencing of Gateway compatible attL1-attR5 entry clones |
| rbcS-E9t-R | tagaggccacgatttgacacattt | Oligo for Sanger sequencing of Gateway compatible attL1-attR5 entry clones |
| CGBE1-gBk-Seq1 | CCACGGTTACCTGGAATCAT | Oligo for Sanger sequencing of Gateway compatible attL1-attR5 entry clones |
| rXRCC1-Seq1 | GAAATCAGCCTTCGCCATGT | Oligo for Sanger sequencing of Gateway compatible attL1-attR5 entry clones |
| rXRCC1-Seq2 | GAATAAACAAGGCGGCAAGC | Oligo for Sanger sequencing of Gateway compatible attL1-attR5 entry clones |
| rXRCC1-Seq3 | GTTATCGGATGAGAAGACGC | Oligo for Sanger sequencing of Gateway compatible attL1-attR5 entry clones |
| M13-R1 | TTTGAGACACGGGCCAGAGCTGC | Oligo for Sanger sequencing of Gateway compatible attL5-attL2 entry clones |
| CGBE1-gBk | atgcttttttataatgccaactttgtacaaaaaagcaggctccgaattcgcccttcATGAAAAGAACTGCAGATGGATCCGAGTTCGAGTCCCCGAAAAAGAAACGGAAAGTGGCAAATGAGCTTACATGGCATGATGTGCTCGCGGAAGAAAAGCAGCAGCCTTATTTCCTGAATACGCTTCAGACGGTTGCCTCTGAAAGGCAATCTGGAGTTACTATATATCCGCCACAGAAGGATGTGTTTAATGCTTTCAGATTCACGGAACTGGGCGACGTGAAGGTGGTGATCTTGGGACAGGACCCCTATCATGGCCCGGGCCAGGCGCATGGCCTCGCCTTTTCTGTTCGGCCGGGGATCGCCATCCCTCCATCTCTCCTGAATATGTATAAAGAATTGGAAAATACAATCCCGGGGTTCACGCGCCCGAACCACGGTTACCTGGAATCATGGGCCAGGCAGGGCGTGCTGCTGCTGAATACGGTGCTGACGGTCCGGGCGGGCCAGGCACATTCTCATGCCTCATTGGGTTGGGAGACGTTTACTGACAAGGTGATAAGCCTGATAAACCAGCATCGGGAGGGAGTCGTTTTCCTTCTTTGGGGATCTCATGCACAGAAGAAGGGAGCCATCATAGACAAACAGAGACACCACGTCTTGAAGGCACCGCATCCCTCACCTCTCTCTGCCCACCGGGGTTTTTTTGGGTGTAACCACTTCGTCTTGGCAAATCAGTGGTTGGAACAGAGAGGGGAAACTCCTATCGATTGGATGCCGGTCCTGCCGGCAGAATCCGAATCCGGGGGTTCGGGGGGAAGTGGCGGGTCTTCCTCTGAGACGGGACCCGTTGCTGTTGACCCTACCCTGAGGCGCAGAATTGAACCCCACGAATTCGAAGTCTTCTTCGACCCACGGGAACTTGCTAAGGAAACGTGTCTGCTGTATGAGATCAACTGGGGAGGCAGACATTCTATTTGGAGGCACACAAGCCAAAATACTAATAAGCACGTTGAGGTGAACTTTATAGAAAAATTTACAACAGAAAGATACTTTTGTCCTAACACAAGGTGTTCAATAACTTGGTTCCTGAGTTGGTCACCCTGCGGCGAATGTTCGCGCGCGATAACCGAATTCCTTTCCCGCTACCCACATGTCACTCTCTTTATTTATATTGCCCGGCTCTACCACCACGCCGATCCGAGGAACAGACAGGGACTCCGGGACCTGATCTCAAGCGGCGTTACTATACAGATTATGACAGAGCAGGAATCCGGTTATTGCTGGCGCAACTTTGTTAATTACTCGCCCTCAAATGAGGCGCACTGGCCACGGTACCCACATTTGTGGGTCCGCTTGTATGTGTTGGAGCTGTATTGCATCATACTTGGCTTGCCACCCTGTCTTAACATACTCCGCCGGAAGCAGCCTCAGCTCACGTTTTTTACAATCGCTCTGCAAAGCTGTCATTATCAGAGACTTCCGCCACATATACTGTGGGCTACTGGGTTGAAGtcaggttctgagacccctggcacaagtgagtcagcaacacccgagtccaccatggattaca | gBlock™ to prepare pYPQ265K and pYPQ265L2 |
| UGN-gBk | ATGTTTGGAGAGAGCTGGAAGAAGCACCTCAGCGGGGAGTTCGGGAAACCGTATTTTATCAAGCTAATGGGATTTGTTGCAGAAGAAAGAAAGCATTACACTGTTTATCCACCCCCACACCAAGTCTTCACCTGGACCCAGATGTGTGACATAAAAGATGTGAAGGTTGTCATCCTGGGACAGGATCCATATCATGGACCTAATCAAGCTCACGGGCTCTGCTTTAGTGTTCAAAGACCTGTTCCGCCTCCGCCCAGTTTGGAGAACATTTACAAAGAGTTGTCTACAGACATAGAGGATTTTGTTCATCCTGGCCACGGAGATTTATCTGGGTGGGCCAAGCAAGGTGTTCTCCTTCTCAACGCTGTCCTCACGGTTCGTGCCCATCAAGCCAACTCTCATAAGGAGCGAGGCTGGGAGCAGTTCACTGATGCAGTTGTGTCCTGGCTAAATCAGAACTCGAATGGCCTTGTTTTCTTGCTCTGGGGCTCTTATGCTCAGAAGAAGGGCAGTGCCATTGATAGGAAGCGGCACCATGTACTACAGACGGCTCATCCCTCCCCTTTGTCAGTGTATAGAGGGTTCTTTGGATGTAGACACTTTTCAAAGACCAATGAGCTGCTGCAGAAGTCTGGCAAGAAGCCCATTGACTGGAAGGAGCTG | gBlock™ to prepare pYPQ265N1 and pYPQ265N2 |
| rXRCC1-gBk | CCTGAAATCAGCCTTCGCCATGTGGTGTCATGCTCCTCGCAAGACTCAACGCATCGGGCCGAAAACCTCTTGAAGGCGGACACATACCGCAAGTGGAGGTCAGCAAAAGCAGGCGAGAAGACGATATCGGTGGTCCTTCAGCTTGAAAAAGAGGAGCAAATACATTCTGTCGACATCGGGAATGACGGCAGTGCCTTTGTCGAGGTTCTTGTTGGGTCATCAGCGGGCGGAGCAACAGCTGGCGAACAGGACTACGAGGTCCTGTTGGTCACCAGCAGCTTTATGAGCCCTAGTGAGTCAAGAAGTGGATCTAATCCTAATAGGGTCAGAATCTTTGGCCCAGACAAGCTCGTCAGAGCAGCGGCAGAGAAGAGGTGGGACAGAGTCAAGATCGTTTGTTCGCAACCGTATTCCAAGGACTCACCGTATGGTCTTTCTTTCGTTAAATTTCACTCCCCGCCAGACAAAGATGAGGCAGAAGCGCCCTCCCAGAAAGTGACCGTGACGAAGCTGGGCCAGTTCCGCGTGAAGGAGGAAGACGACTCTGCGAACTCCCTCCGGCCAGGGGCACTTTTTTTCAATAGAATAAACAAGGCGGCAAGCGCAAGCGCGTCGGACCCCGCTGGCCCGAGCTACGCTGCAGCGACTCTTCAAGCGTCTTCAGCAGCAAGCTCAGCTTTGCCAGTCCCAAAGGTTGGCGGCTCGAGCAGCAAACTTCAAGAGCCCCCGAAGGGCAAGCGCAAGCTGGACCTGGGGCTCGAAGATTCTAAGCCCCCCTCCAAGCCTAGCGCAGGTCCGGCAGCTCTCAAGCGGCCTAAGCTGCCAGTTCCTTCTAGGACCCCTGCTGCAACACCCGCAAGTACACCGGCGCAGAAGGCAGTCCCCGGAAAACCAAGGGGTGAGGGGACCGAGCCACGGGGGGCTCGGGCGGGTCCTCAAGAACTTGGAAAAATATTGCAGGGGGTCGTCGTTGTGCTGTCGGGGTTTCAAAACCCGTTCCGGTCAGAGCTGAGAGACAAGGCGCTGGAATTGGGGGCGAAATACAGACCGGATTGGACGCCAGATAGTACTCACTTGATATGTGCATTTGCAAACACTCCTAAGTACTCTCAGGTTTTGGGGCTTGGTGGTCGGATTGTTCGGAAAGAATGGGTGCTGGACTGTTATCGGATGAGAAGACGCCTTCCTTCTCAACGGTATCTTATGGCCGGGTTGGGTAGTAGCTCAGAAGACGAGGGCGATAGCCACTCTGAGTCGGGAGAGGACGAGGCGCCAAAGCTCCCTAGAAAGAGGCCCCAACCTAAGGCCAAAACACAGGCCGCTGGCCCGAGTTCACCCCCCAGACCCCCAACCCCCGAGGAAACAAAAGCGCCTTCCCCTGGACCGCAGGACAACAGCGACACAGATGGCGAGCAGTCCGAAGGCCGCGATAATGGTGCTGAAGACTCGGGAGACACAGAAGACGAGCTTCGGCGCGTTGCAAAACAGAGAGAACAGAGGCAGCCACCGGCACCCGAAGAAAATGGTGAGGATCCTTATGCGGGCTCAACAGATGAGAATACGGACAGTGAGGCGCCATCTGAGGCCGATCTGCCAATACCAGAGCTCCCCGATTTTTTCCAAGGCAAGCACTTTTTTCTGTACGGTGAATTCCCAGGCGACGAACGCAGGAAGCTTATTAGGTATGTCACCGCTTTCAACGGGGAGTTGGAAGACTACATGAGCGACCGCGTCCAGTTCGTGATAACAGCCCAGGAGTGGGACCCTAACTTTGAAGAGGCATTGATGGAAAACCCTTCGTTGGCATTCGTGAGGCCCAGATGGATCTACAGTTGTAATGAGAAGCAAAAGCTGTTGCCACATCAACTTTACGGCGTTGTTCCGCAGGCG | gBlock™ to prepare pYPQ265O1 and pYPQ265O2 |
|  |  |  |

**Supplementary Table 2. T-DNA constructs used in this study**

| **Construct** | **Purpose** | **Figure shown** |
| --- | --- | --- |
| **pLR3741** | For C-to-G base editing at OsALS-sgRNA32 site in rice | Figure 1, 4; Supplementary Figure 1, 2, 3, 10 |
| **pLR3742** | For C-to-G base editing at OsCGRS55-sgRNA-sgRNA site in rice | Figure 1; Supplementary Figure 1, 2, 3 |
| **pLR3744** | For C-to-G base editing at OsALS-sgRNA32 site in rice | Figure 1, 4; Supplementary Figure 1, 2, 3 |
| **pLR3745** | For C-to-G base editing at OsCGRS55-sgRNA-sgRNA site in rice | Figure 1; Supplementary Figure 1, 2, 3 |
| **pLR3747** | For C-to-G base editing at OsALS-sgRNA32 site in rice | Figure 1, 4; Supplementary Figure 1, 2, 3, 10 |
| **pLR3748** | For C-to-G base editing at OsCGRS55-sgRNA-sgRNA site in rice | Figure 1; Supplementary Figure 1, 2, 3 |
| **pLR3750** | For C-to-G base editing at OsALS-sgRNA32 site in rice | Figure 1, 4; Supplementary Figure 1, 2, 3, 10 |
| **pLR3751** | For C-to-G base editing at OsCGRS55-sgRNA-sgRNA site in rice | Figure 1; Supplementary Figure 1, 2, 3 |
| **pLR3753** | For C-to-G base editing at OsALS-sgRNA22 site in rice | Figure 3; Supplementary Figure 7, 8, 9 |
| **pLR3754** | For C-to-G base editing at OsALS-sgRNA24 site in rice | Figure 3; Supplementary Figure 7, 8, 9 |
| **pLR3755** | For C-to-G base editing at OsALS-sgRNA32 site in rice | Figure 3, 4; Supplementary Figure 7, 8, 9 |
| **pLR3756** | For C-to-G base editing at OsALS-sgRNA31 site in rice | Figure 3; Supplementary Figure 7, 8, 9 |
| **pLR3758** | For C-to-G base editing at OsALS-sgRNA147 site in rice | Figure 3; Supplementary Figure 7, 8, 9 |
| **pLR3760** | For C-to-G base editing at OsALS-sgRNA150 site in rice | Figure 3; Supplementary Figure 7, 8, 9 |
| **pLR3767** | For C-to-G base editing at OsEPSPS-sgRNA31 site in rice | Figure 3; Supplementary Figure 7, 8, 9 |
| **pLR3768** | For C-to-G base editing at OsEPSPS-sgRNA30 site in rice | Figure 3; Supplementary Figure 7, 8, 9 |
| **pLR3772** | For C-to-G base editing at OsCGRS55-sgRNA-sgRNA site in rice | Figure 3; Supplementary Figure 7, 8, 9 |
| **pLR3773** | For C-to-G base editing at OsALS-sgRNA22 site in rice | Figure 3, 4; Supplementary Figure 7, 8, 9 |
| **pLR3774** | For C-to-G base editing at OsALS-sgRNA24 site in rice | Figure 3; Supplementary Figure 7, 8, 9 |
| **pLR3775** | For C-to-G base editing at OsALS-sgRNA32 site in rice | Figure 3, 4; Supplementary Figure 7, 8, 9 |
| **pLR3776** | For C-to-G base editing at OsALS-sgRNA31 site in rice | Figure 3; Supplementary Figure 7, 8, 9 |
| **pLR3778** | For C-to-G base editing at OsALS-sgRNA147 site in rice | Figure 3; Supplementary Figure 7, 8, 9 |
| **pLR3780** | For C-to-G base editing at OsALS-sgRNA150 site in rice | Figure 3; Supplementary Figure 7, 8, 9 |
| **pLR3787** | For C-to-G base editing at OsEPSPS-sgRNA31 site in rice | Figure 3; Supplementary Figure 7, 8, 9 |
| **pLR3788** | For C-to-G base editing at OsEPSPS-sgRNA30 site in rice | Figure 3; Supplementary Figure 7, 8, 9 |
| **pLR3792** | For C-to-G base editing at OsCGRS55-sgRNA-sgRNA site in rice | Figure 3; Supplementary Figure 7, 8, 9 |
| **pLR3793** | For C-to-G base editing at OsALS-sgRNA22 site in rice | Figure 3, 4; Supplementary Figure 7, 8, 9 |
| **pLR3794** | For C-to-G base editing at OsALS-sgRNA24 site in rice | Figure 3; Supplementary Figure 7, 8, 9 |
| **pLR3795** | For C-to-G base editing at OsALS-sgRNA32 site in rice | Figure 3, 4; Supplementary Figure 7, 8, 9 |
| **pLR3796** | For C-to-G base editing at OsALS-sgRNA31 site in rice | Figure 3; Supplementary Figure 7, 8, 9 |
| **pLR3798** | For C-to-G base editing at OsALS-sgRNA147 site in rice | Figure 3; Supplementary Figure 7, 8, 9 |
| **pLR3800** | For C-to-G base editing at OsALS-sgRNA150 site in rice | Figure 3; Supplementary Figure 7, 8, 9 |
| **pLR3807** | For C-to-G base editing at OsEPSPS-sgRNA31 site in rice | Figure 3; Supplementary Figure 7, 8, 9 |
| **pLR3808** | For C-to-G base editing at OsEPSPS-sgRNA30 site in rice | Figure 3; Supplementary Figure 7, 8, 9 |
| **pLR3812** | For C-to-G base editing at OsCGRS55-sgRNA-sgRNA site in rice | Figure 3; Supplementary Figure 7, 8, 9 |
| **pLR4023** | For C-to-G base editing at PDS1-sgRNA8 site in poplar | Figure 5 |
| **pLR4024** | For C-to-G base editing at PDS1-sgRNA8 site in poplar | Figure 5 |
| **pLR4025** | For C-to-G base editing at PDS1-sgRNA8 site in poplar | Figure 5 |
| **pLR4026** | For C-to-G base editing at PDS1-sgRNA9 site in poplar | Figure 5 |
| **pLR4027** | For C-to-G base editing at PDS1-sgRNA9 site in poplar | Figure 5 |
| **pLR4028** | For C-to-G base editing at PDS1-sgRNA9 site in poplar | Figure 5 |
| **pLR4037** | For C-to-G base editing at Sago7-gR1 in tomato | Figure 2; Supplementary Figure 4, 5, 6 |
| **pLR4038** | For C-to-G base editing at Sago7-gR1 in tomato | Figure 2; Supplementary Figure 4, 5, 6 |
| **pLR4039** | For C-to-G base editing at Sago7-gR1 in tomato | Figure 2; Supplementary Figure 4, 5, 6 |
| **pLR4040** | For C-to-G base editing at Sago7-gR1 in tomato | Figure 2; Supplementary Figure 4, 5, 6 |
| **pLR4041** | For C-to-G base editing at Sago7-gR2 in tomato | Figure 2; Supplementary Figure 4, 5, 6 |
| **pLR4042** | For C-to-G base editing at Sago7-gR2 in tomato | Figure 2; Supplementary Figure 4, 5, 6 |
| **pLR4043** | For C-to-G base editing at Sago7-gR2 in tomato | Figure 2; Supplementary Figure 4, 5, 6 |
| **pLR4044** | For C-to-G base editing at Sago7-gR2 in tomato | Figure 2; Supplementary Figure 4, 5, 6 |
| **pLR4045** | For C-to-G base editing at Sago7-gR3 in tomato | Figure 2; Supplementary Figure 4, 5, 6 |
| **pLR4046** | For C-to-G base editing at Sago7-gR3 in tomato | Figure 2; Supplementary Figure 4, 5, 6 |
| **pLR4047** | For C-to-G base editing at Sago7-gR3 in tomato | Figure 2; Supplementary Figure 4, 5, 6 |
| **pLR4048** | For C-to-G base editing at Sago7-gR3 in tomato | Figure 2; Supplementary Figure 4, 5, 6 |
| **pLR4049** | For C-to-G base editing at Sago7-gR4 in tomato | Figure 2; Supplementary Figure 4, 5, 6 |
| **pLR4050** | For C-to-G base editing at Sago7-gR4 in tomato | Figure 2; Supplementary Figure 4, 5, 6 |
| **pLR4051** | For C-to-G base editing at Sago7-gR4 in tomato | Figure 2; Supplementary Figure 4, 5, 6 |
| **pLR4052** | For C-to-G base editing at Sago7-gR4 in tomato | Figure 2; Supplementary Figure 4, 5, 6 |
|  |  |  |
