# Supplementary material for: Exploring C-To-G Base Editing in Rice, Tomato, and Poplar
Source: Front Genome Ed. 2021 Sep 15;3:756766. doi: 10.3389/fgeed.2021.756766 (PMC8525388; doi:10.3389/fgeed.2021.756766)
Supplement: Supplementary file 2 [file Presentation1.PPTX]

## Slide 1
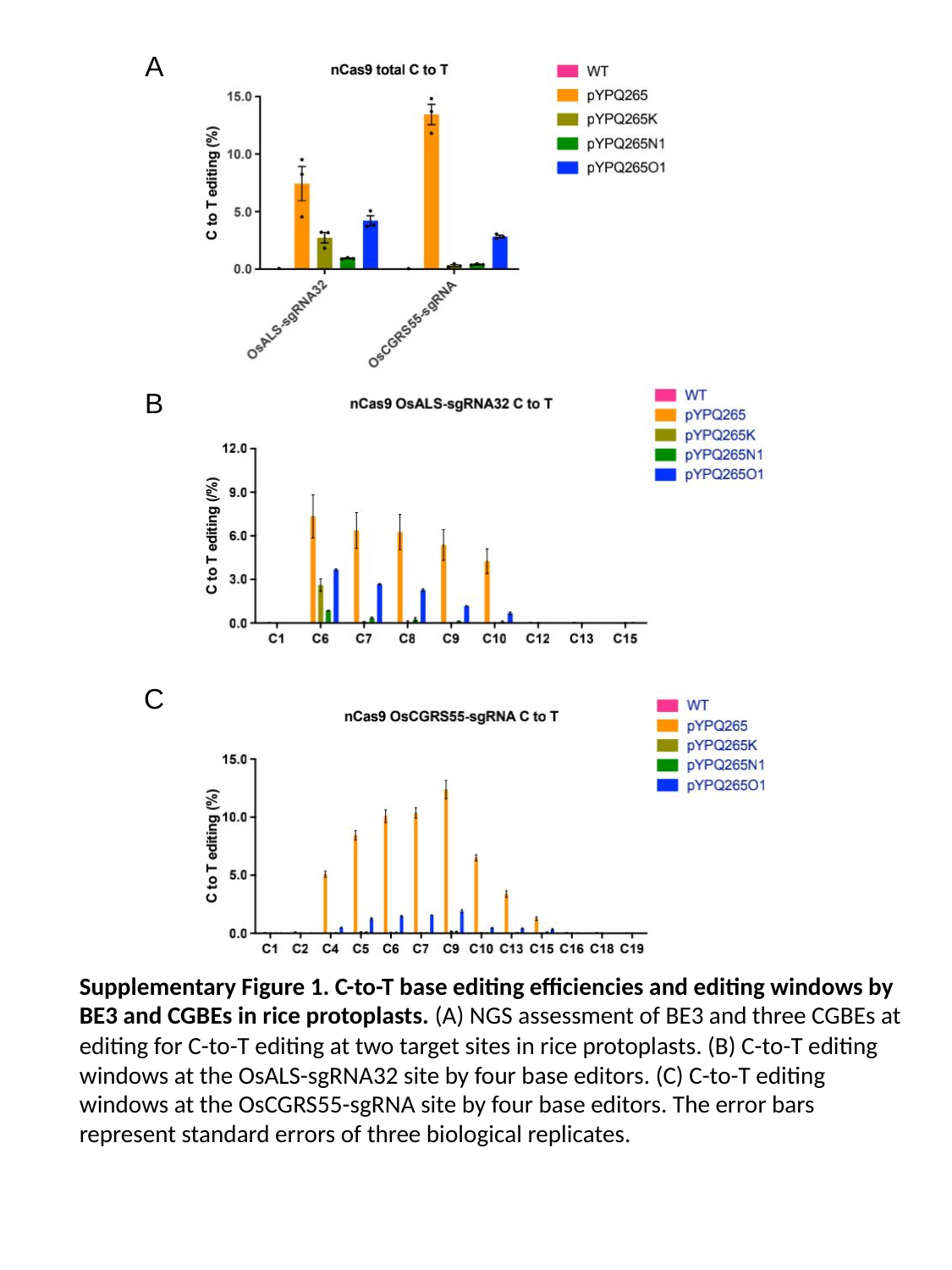

A
B
C
Supplementary Figure 1. C-to-T base editing efficiencies and editing windows by BE3 and CGBEs in rice protoplasts. (A) NGS assessment of BE3 and three CGBEs at editing for C-to-T editing at two target sites in rice protoplasts. (B) C-to-T editing windows at the OsALS-sgRNA32 site by four base editors. (C) C-to-T editing windows at the OsCGRS55-sgRNA site by four base editors. The error bars represent standard errors of three biological replicates.

## Slide 2
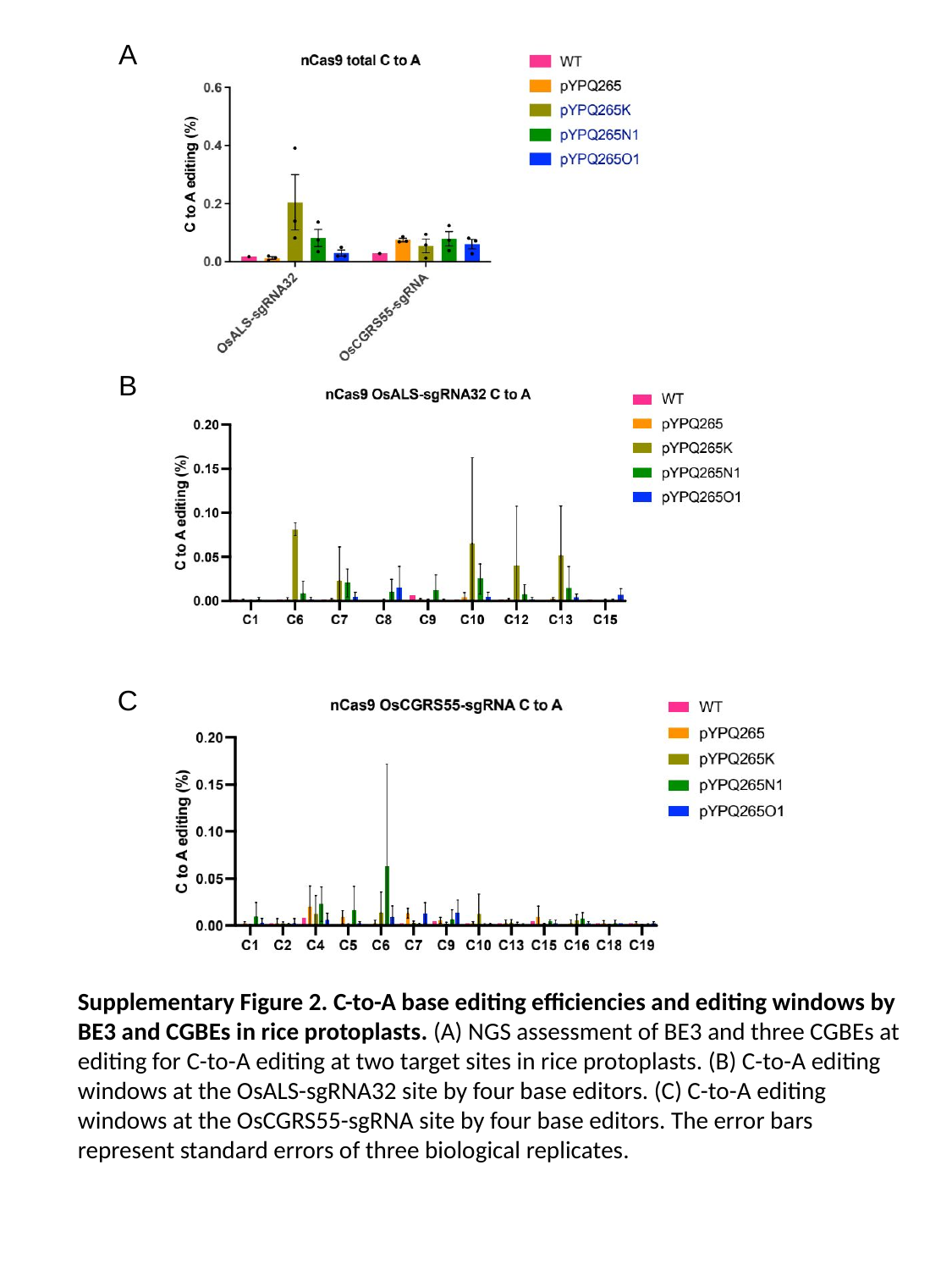

A
B
C
Supplementary Figure 2. C-to-A base editing efficiencies and editing windows by BE3 and CGBEs in rice protoplasts. (A) NGS assessment of BE3 and three CGBEs at editing for C-to-A editing at two target sites in rice protoplasts. (B) C-to-A editing windows at the OsALS-sgRNA32 site by four base editors. (C) C-to-A editing windows at the OsCGRS55-sgRNA site by four base editors. The error bars represent standard errors of three biological replicates.

## Slide 3
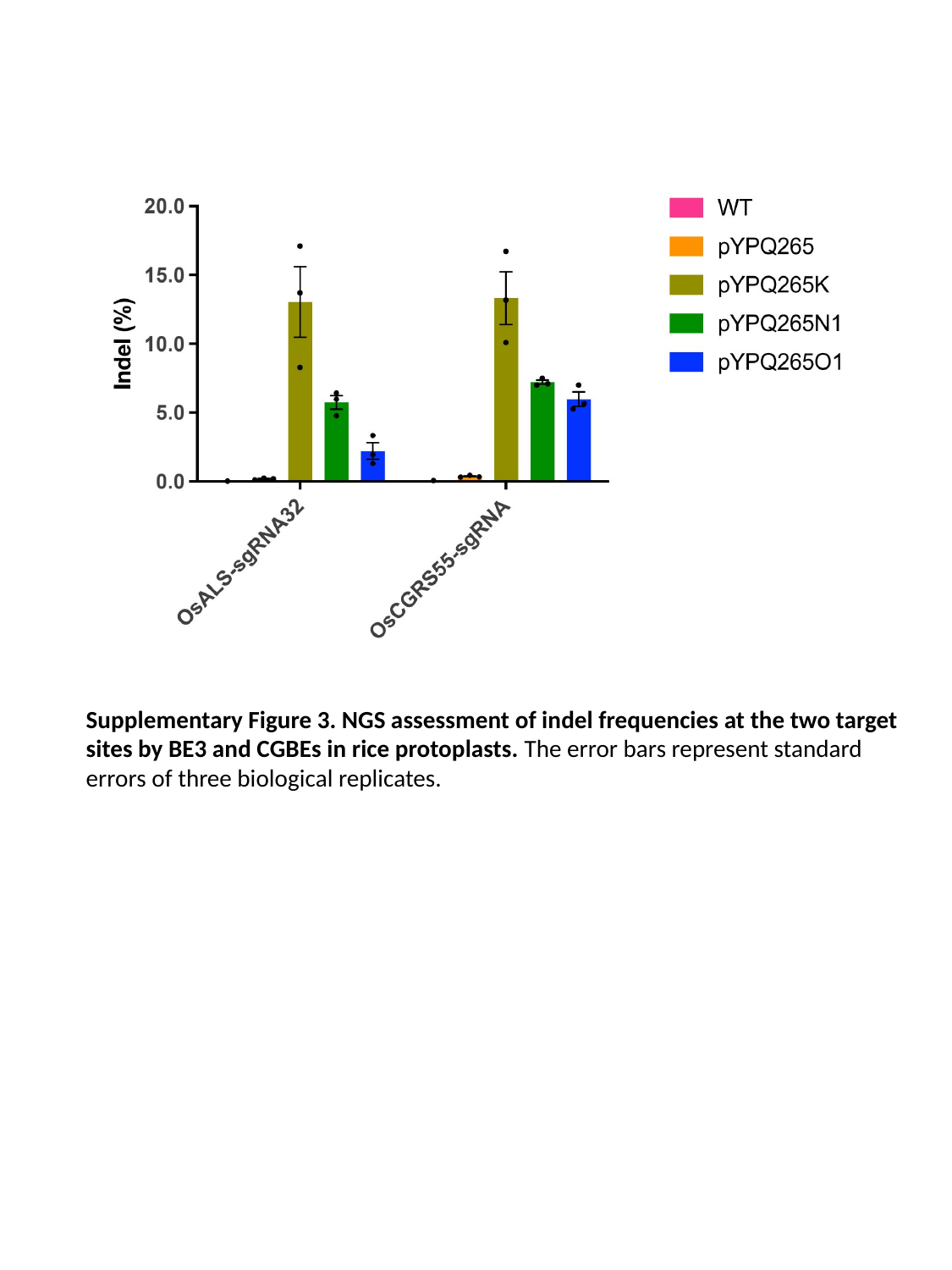

Supplementary Figure 3. NGS assessment of indel frequencies at the two target sites by BE3 and CGBEs in rice protoplasts. The error bars represent standard errors of three biological replicates.

## Slide 4
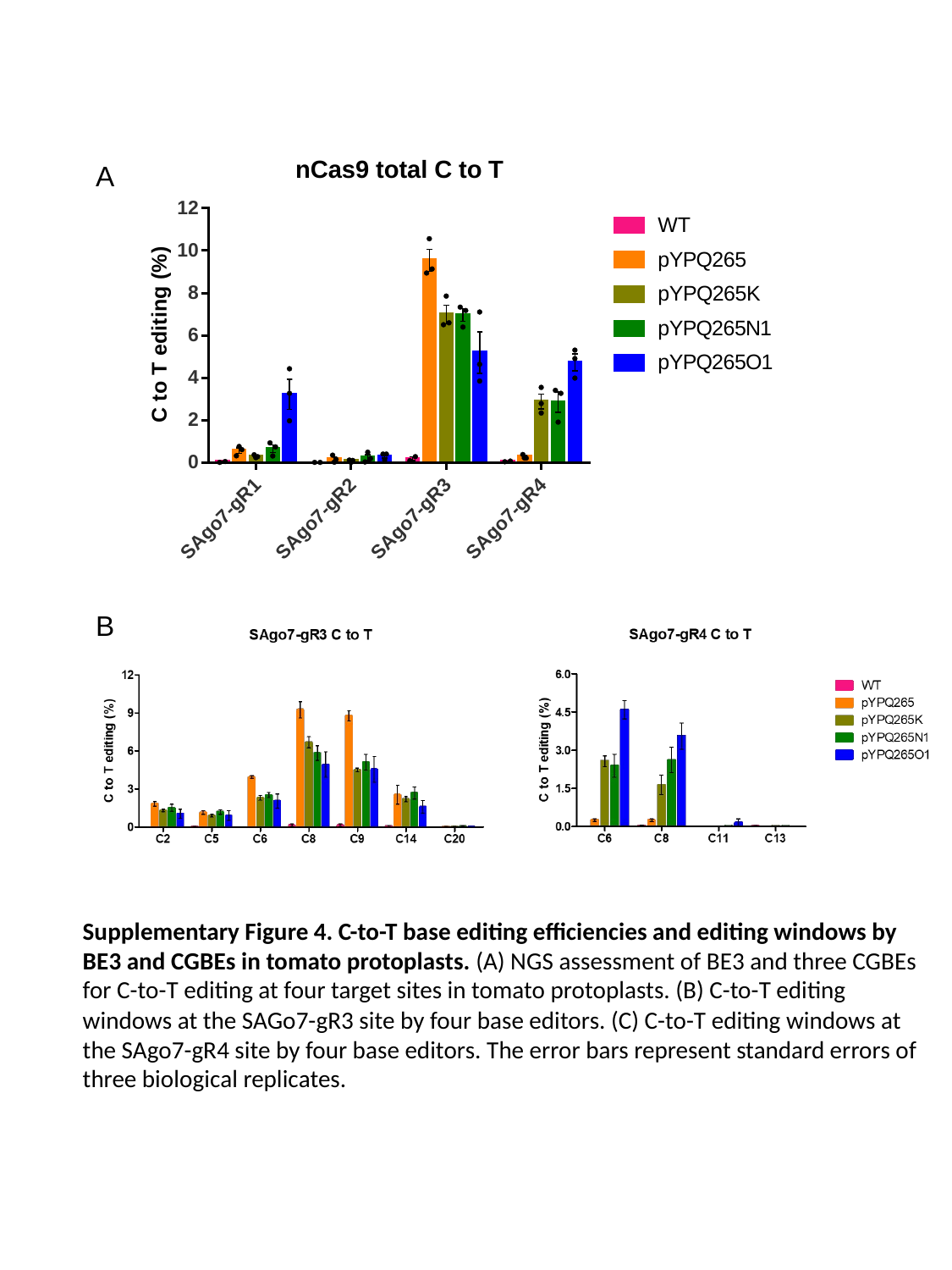

A
B
Supplementary Figure 4. C-to-T base editing efficiencies and editing windows by BE3 and CGBEs in tomato protoplasts. (A) NGS assessment of BE3 and three CGBEs for C-to-T editing at four target sites in tomato protoplasts. (B) C-to-T editing windows at the SAGo7-gR3 site by four base editors. (C) C-to-T editing windows at the SAgo7-gR4 site by four base editors. The error bars represent standard errors of three biological replicates.

## Slide 5
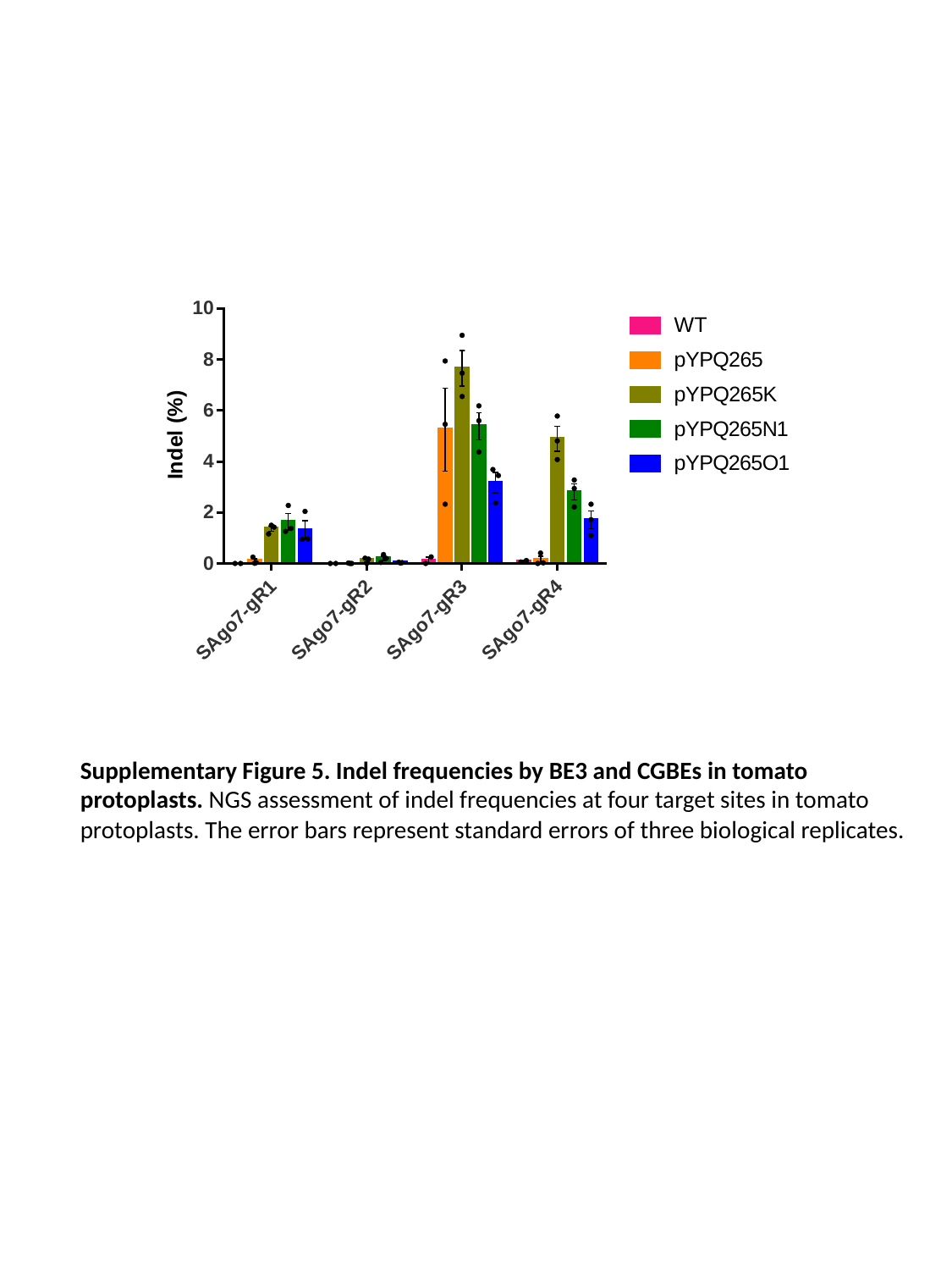

Supplementary Figure 5. Indel frequencies by BE3 and CGBEs in tomato protoplasts. NGS assessment of indel frequencies at four target sites in tomato protoplasts. The error bars represent standard errors of three biological replicates.

## Slide 6
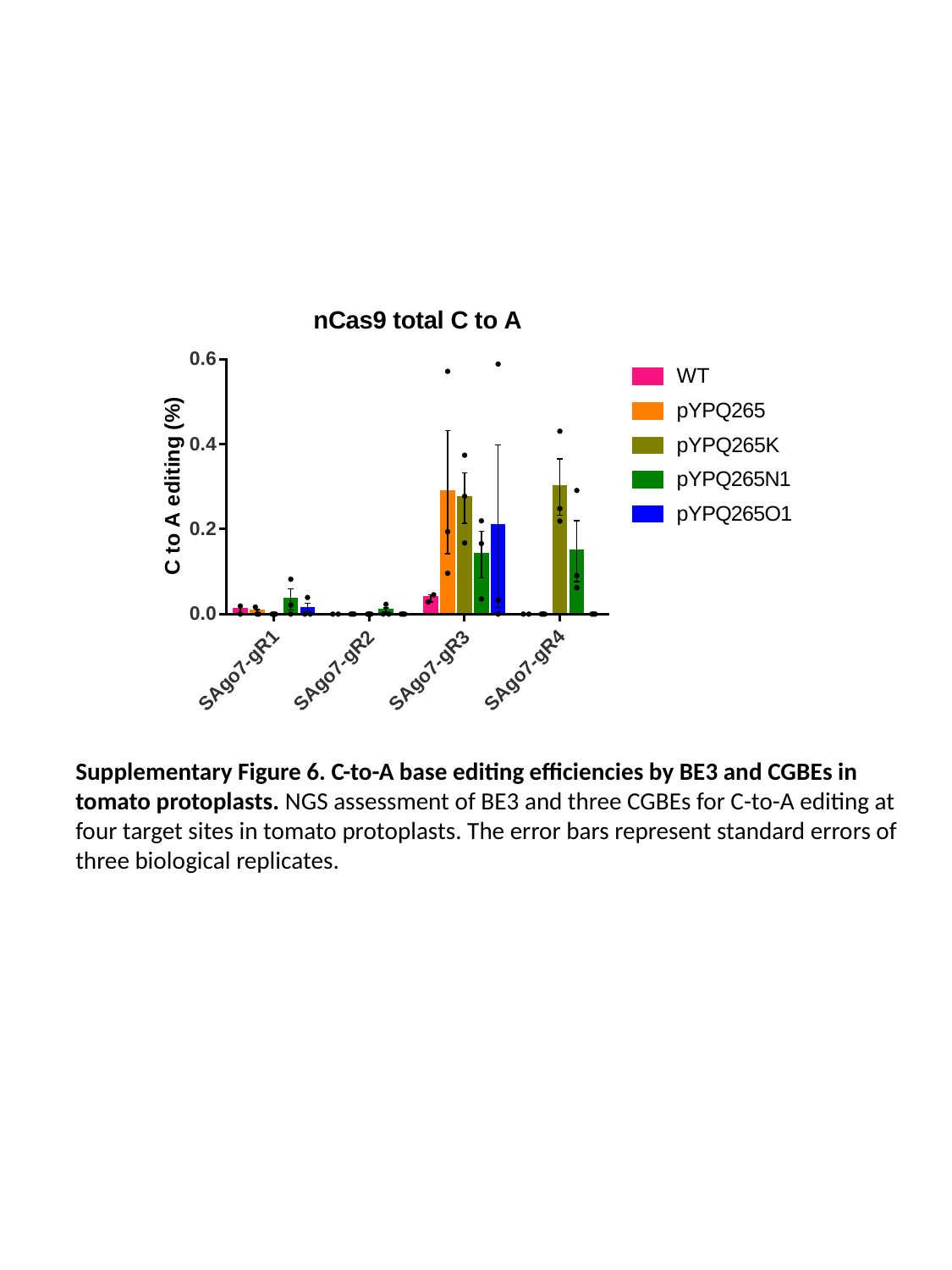

Supplementary Figure 6. C-to-A base editing efficiencies by BE3 and CGBEs in tomato protoplasts. NGS assessment of BE3 and three CGBEs for C-to-A editing at four target sites in tomato protoplasts. The error bars represent standard errors of three biological replicates.

## Slide 7
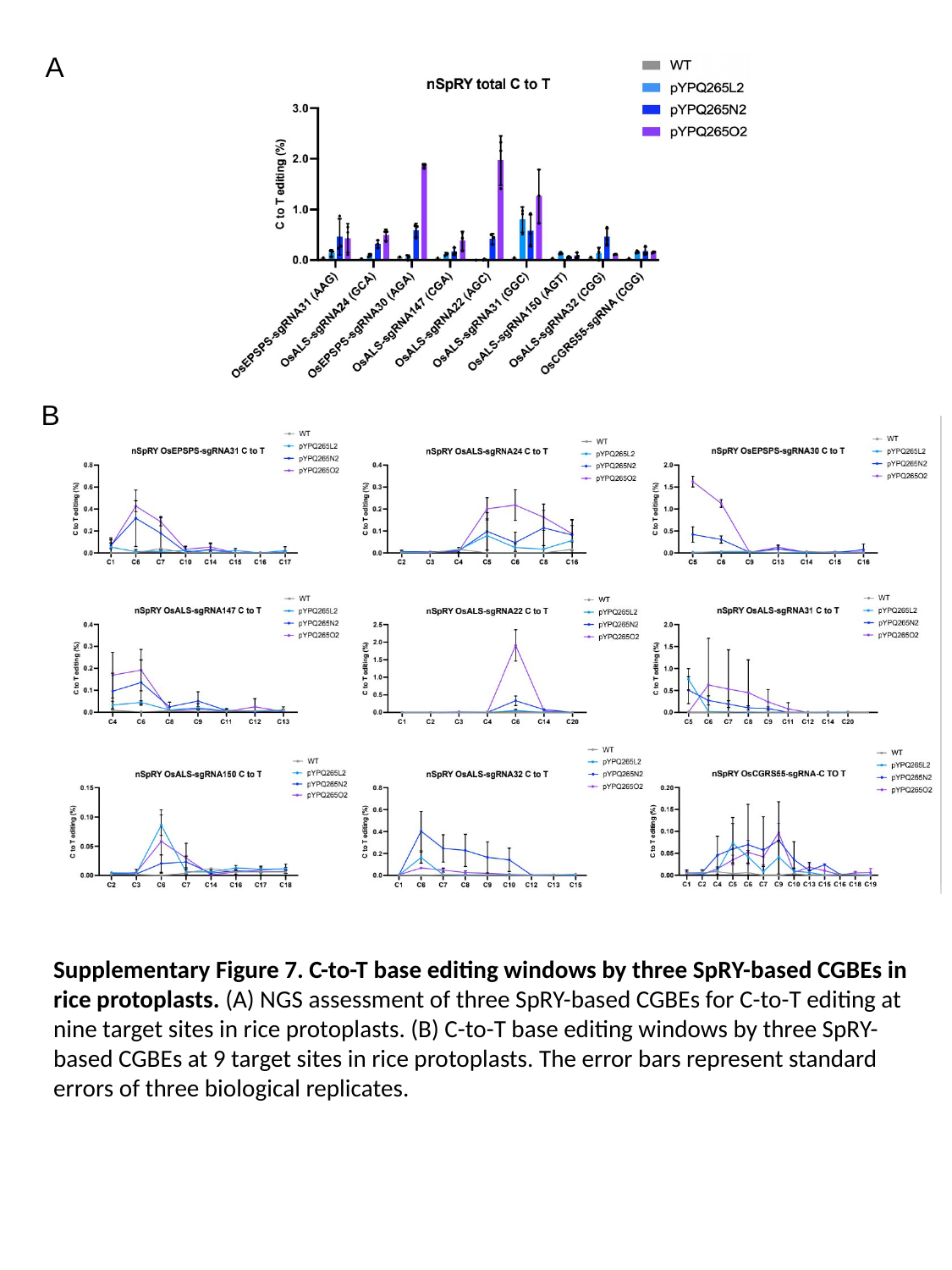

A
B
Supplementary Figure 7. C-to-T base editing windows by three SpRY-based CGBEs in rice protoplasts. (A) NGS assessment of three SpRY-based CGBEs for C-to-T editing at nine target sites in rice protoplasts. (B) C-to-T base editing windows by three SpRY-based CGBEs at 9 target sites in rice protoplasts. The error bars represent standard errors of three biological replicates.

## Slide 8
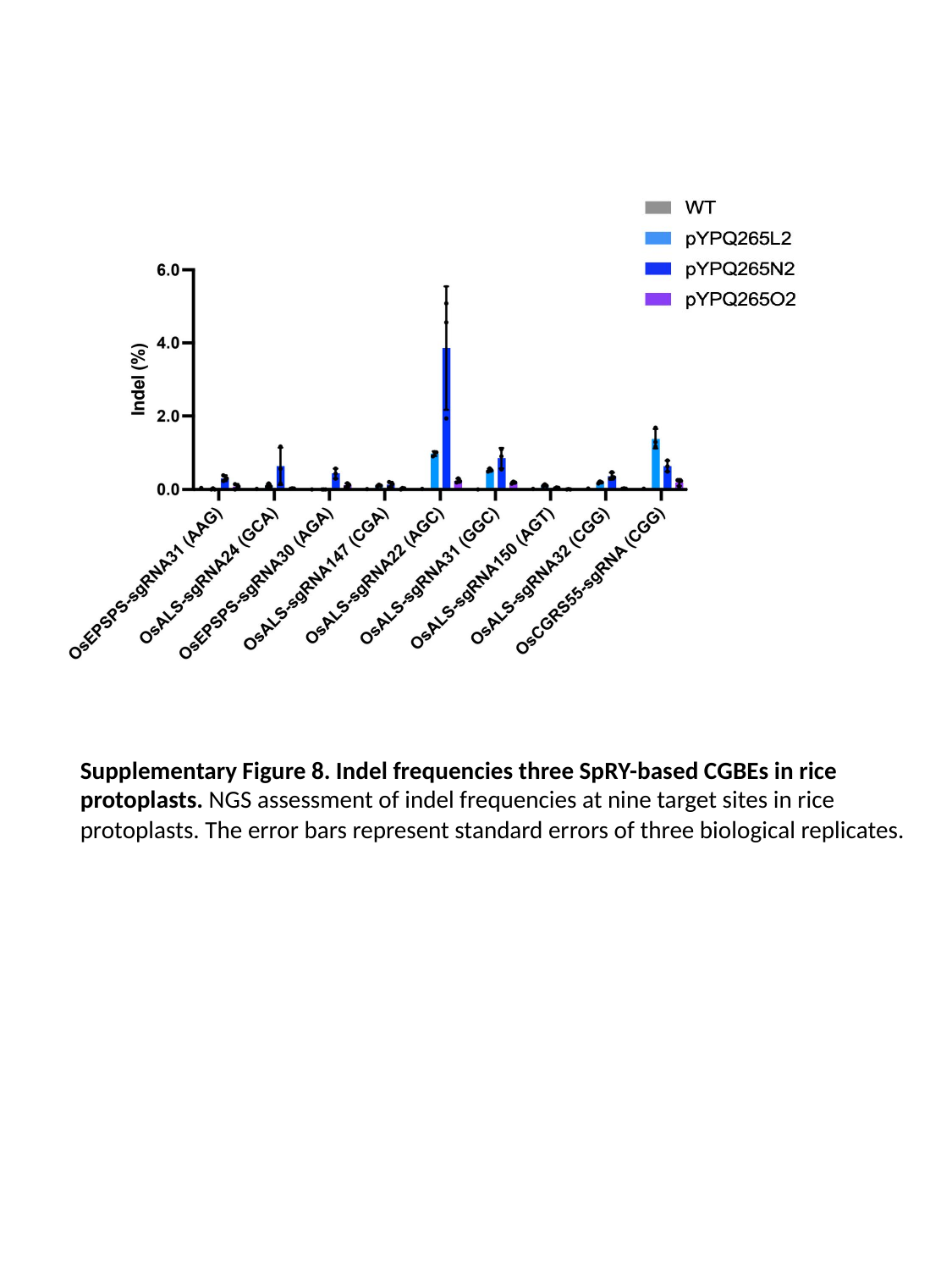

Supplementary Figure 8. Indel frequencies three SpRY-based CGBEs in rice protoplasts. NGS assessment of indel frequencies at nine target sites in rice protoplasts. The error bars represent standard errors of three biological replicates.

## Slide 9
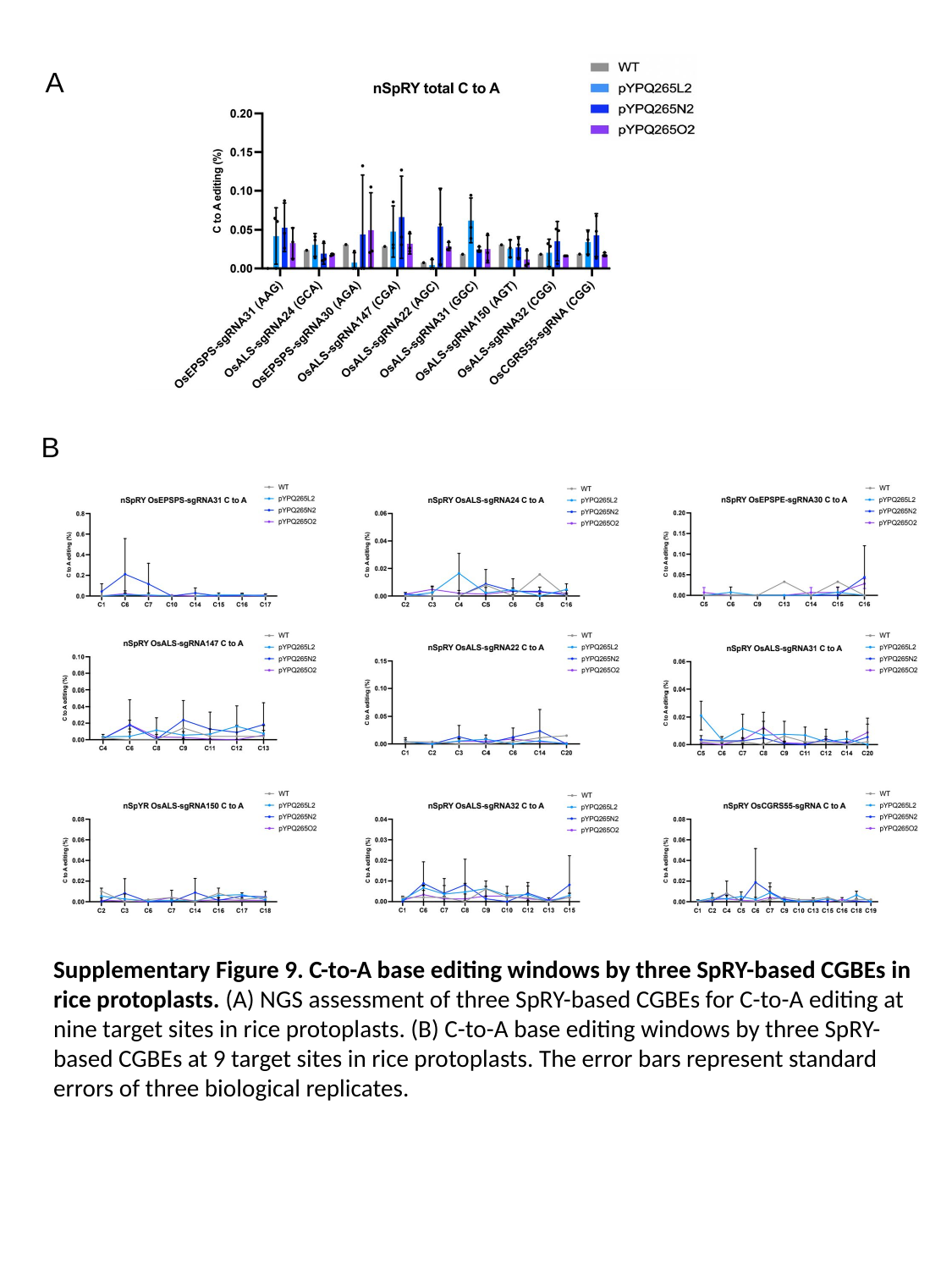

A
B
Supplementary Figure 9. C-to-A base editing windows by three SpRY-based CGBEs in rice protoplasts. (A) NGS assessment of three SpRY-based CGBEs for C-to-A editing at nine target sites in rice protoplasts. (B) C-to-A base editing windows by three SpRY-based CGBEs at 9 target sites in rice protoplasts. The error bars represent standard errors of three biological replicates.

## Slide 10
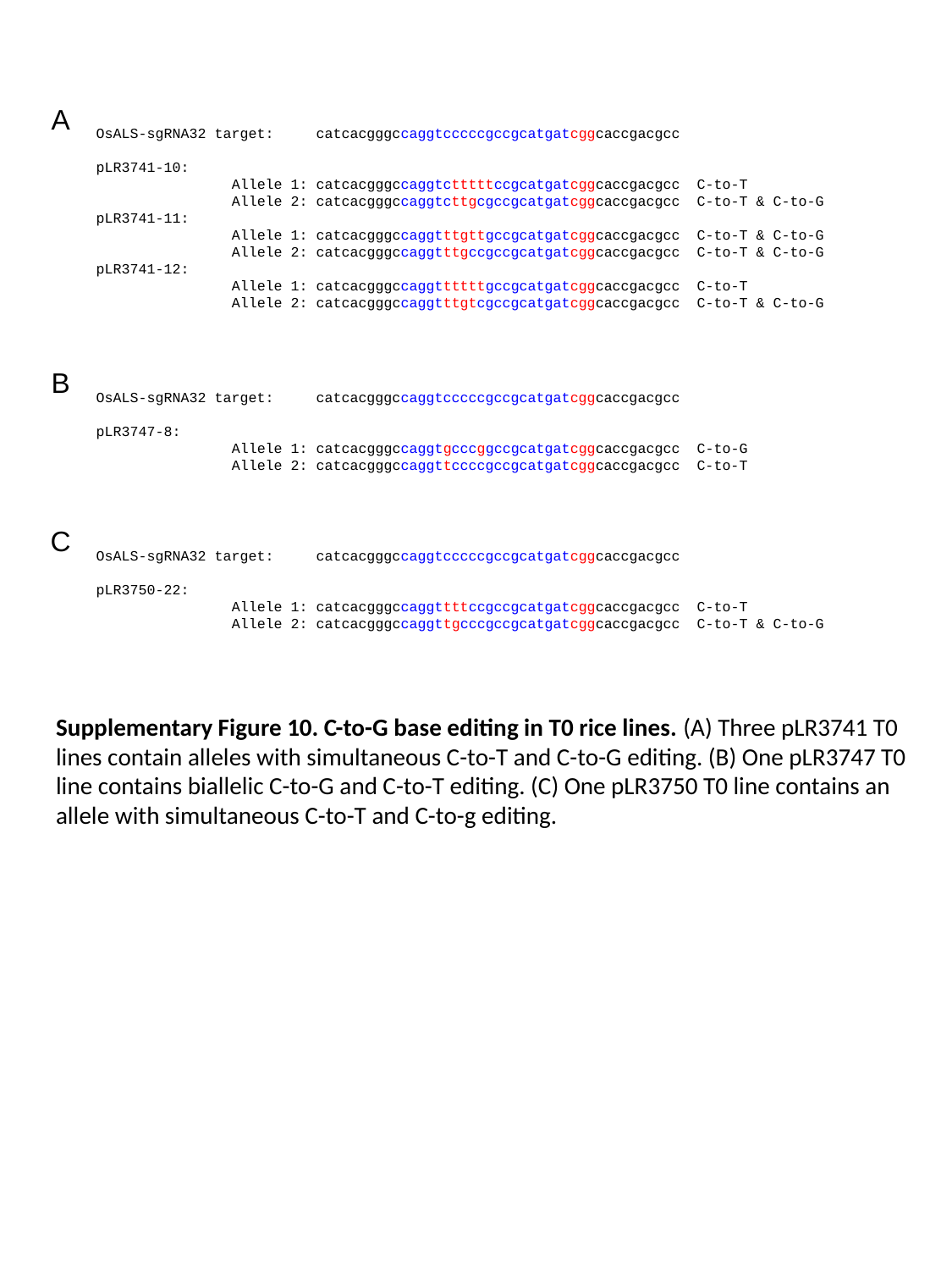

A
OsALS-sgRNA32 target: catcacgggccaggtcccccgccgcatgatcggcaccgacgcc
pLR3741-10:
 Allele 1: catcacgggccaggtctttttccgcatgatcggcaccgacgcc C-to-T
 Allele 2: catcacgggccaggtcttgcgccgcatgatcggcaccgacgcc C-to-T & C-to-G
pLR3741-11:
 Allele 1: catcacgggccaggtttgttgccgcatgatcggcaccgacgcc C-to-T & C-to-G
 Allele 2: catcacgggccaggtttgccgccgcatgatcggcaccgacgcc C-to-T & C-to-G
pLR3741-12:
 Allele 1: catcacgggccaggttttttgccgcatgatcggcaccgacgcc C-to-T
 Allele 2: catcacgggccaggtttgtcgccgcatgatcggcaccgacgcc C-to-T & C-to-G
B
OsALS-sgRNA32 target: catcacgggccaggtcccccgccgcatgatcggcaccgacgcc
pLR3747-8:
 Allele 1: catcacgggccaggtgcccggccgcatgatcggcaccgacgcc C-to-G
 Allele 2: catcacgggccaggttccccgccgcatgatcggcaccgacgcc C-to-T
C
OsALS-sgRNA32 target: catcacgggccaggtcccccgccgcatgatcggcaccgacgcc
pLR3750-22:
 Allele 1: catcacgggccaggttttccgccgcatgatcggcaccgacgcc C-to-T
 Allele 2: catcacgggccaggttgcccgccgcatgatcggcaccgacgcc C-to-T & C-to-G
Supplementary Figure 10. C-to-G base editing in T0 rice lines. (A) Three pLR3741 T0 lines contain alleles with simultaneous C-to-T and C-to-G editing. (B) One pLR3747 T0 line contains biallelic C-to-G and C-to-T editing. (C) One pLR3750 T0 line contains an allele with simultaneous C-to-T and C-to-g editing.
